# The relevance of genetic structure in ecotype designation and conservation management

**DOI:** 10.1111/eva.13339

**Published:** 2022-01-19

**Authors:** Astrid V. Stronen, Anita J. Norman, Eric Vander Wal, Paul C. Paquet

**Affiliations:** ^1^ Department of Biology Biotechnical Faculty University of Ljubljana Ljubljana Slovenia; ^2^ Department of Biotechnology and Life Sciences Insubria University Varese Italy; ^3^ Department of Chemistry and Bioscience Aalborg University Aalborg Denmark; ^4^ Department of Fish, Wildlife and Environmental Studies Swedish University of Agricultural Sciences Umeå Sweden; ^5^ Department of Biology Memorial University of Newfoundland St. John’s NL Canada; ^6^ Department of Geography University of Victoria Victoria BC Canada; ^7^ Raincoast Conservation Foundation Sidney BC Canada

**Keywords:** adaptive genetic diversity, animal ecology, anthropogenic, conservation priority, environmental selection, phenotype, population genetic structure

## Abstract

The concept of ecotypes is complex, partly because of its interdisciplinary nature, but the idea is intrinsically valuable for evolutionary biology and applied conservation. The complex nature of ecotypes has spurred some confusion and inconsistencies in the literature, thereby limiting broader theoretical development and practical application. We provide suggestions for how incorporating genetic analyses can ease confusion and help define ecotypes. We approach this by systematically reviewing 112 publications across taxa that simultaneously mention the terms *ecotyp*e, *conservation* and *management*, to examine the current use of the term in the context of conservation and management. We found that most ecotype studies involve fish, mammals and plants with a focus on habitat use, which at 60% was the most common criterion used for categorization of ecotypes. Only 53% of the studies incorporated genetic analyses, and major discrepancies in available genomic resources among taxa could have contributed to confusion about the role of genetic structure in delineating ecotypes. Our results show that the rapid advances in genetic methods, also for nonmodel organisms, can help clarify the spatiotemporal distribution of adaptive and neutral genetic variation and their relevance to ecotype designations. Genetic analyses can offer empirical support for the ecotype concept and provide a timely measure of evolutionary potential, especially in changing environmental conditions. Genetic variation that is often difficult to detect, including polygenic traits influenced by small contributions from several genes, can be vital for adaptation to rapidly changing environments. Emerging ecotypes may signal speciation in progress, and findings from genome‐enabled organisms can help clarify important selective factors driving ecotype development and persistence, and thereby improve preservation of interspecific genetic diversity. Incorporation of genetic analyses in ecotype studies will help connect evolutionary biology and applied conservation, including that of problematic groups such as natural hybrid organisms and urban or anthropogenic ecotypes.

## 1 INTRODUCTION

The existence of intraspecific long‐term population differences among neighbouring habitats suggests the presence of different ecotypes—variants of a species adapted to distinct environments (Turesson, 1922). Even so, the term is defined and used variably (de Bruyn et al., 2013; Lowry, 2012; Morrison, 2012), and the extent to which spatial and functional genetic structure inform or confound the ecotype concept is unresolved (e.g. Menz et al., 2015; Yannic et al., 2017). For populations exhibiting morphological and neutral genetic differentiation, uncertainties about the extent to which the observed phenotypes are determined by genetic or environmental factors may still exist (e.g. Bozchaloyi & Sheidai, 2018). Despite the apparent importance of ecotypes for applied conservation management (Bourret et al., 2020; Chiesa et al., 2014; Wiedmann & Sargeant, 2014), this uncertainty limits broader theoretical development and practical application (Box 1 Part A).

BOX 1  

Part A: Problems with the ecotype concept

1. A lack of consistent ecotype definition limits broader application, and the role of genetic structure in delineating ecotypes currently ranges from all important to unspecified;
2. Current discrepancies between genetic structure and ecotype may, at least in part, reflect the large variability in available genomic resources across taxa, which has hampered investigation of adaptive vs. neutral differentiation.

Part B: A framework for resolution of ecotypes

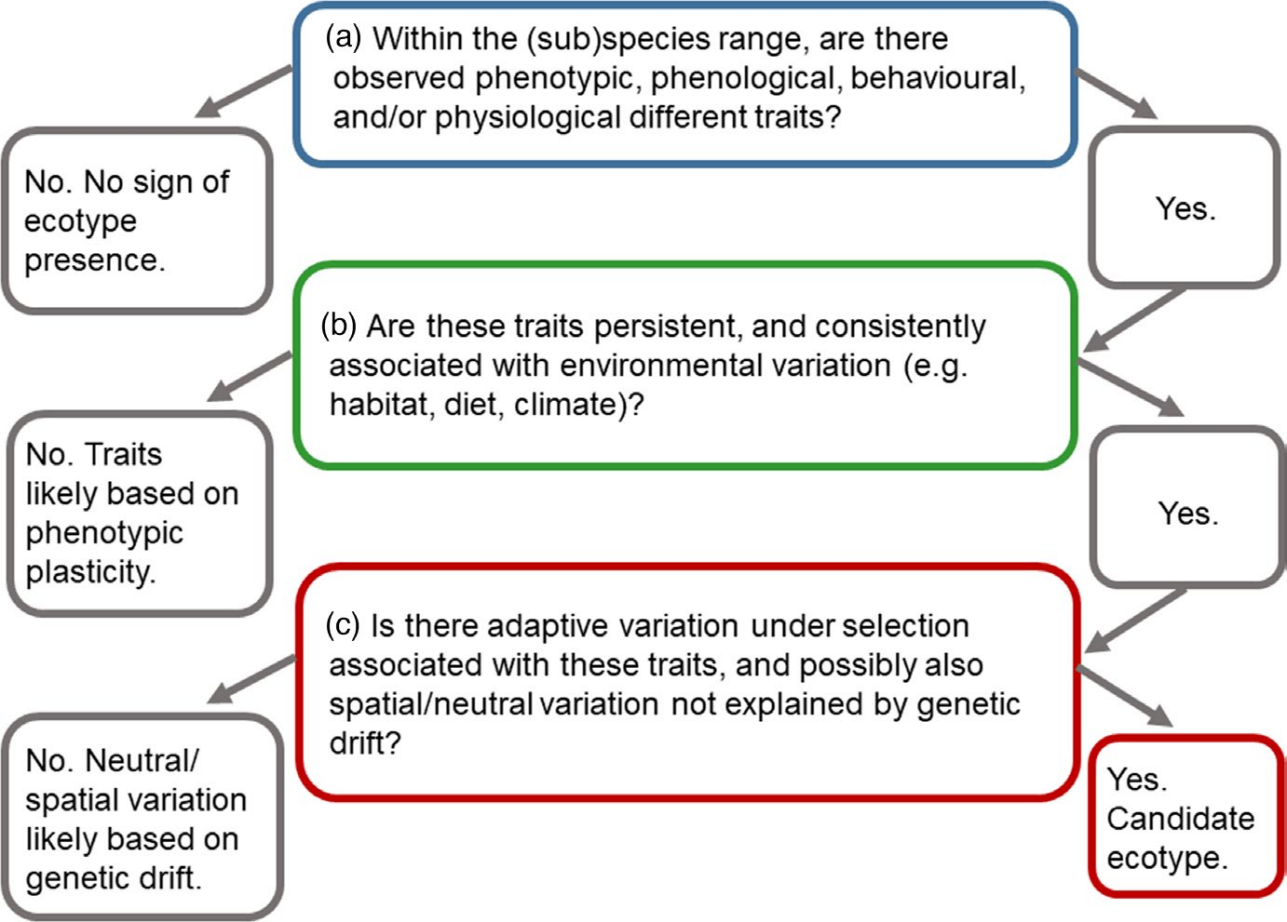

The role of genetic structure remains a major confounding factor for ecotype designations. This includes the question of whether populations that do not show clear evidence of genetic differentiation can be considered as separate ecotypes. Investigations across taxa have emphasized the inherent difficulty in drawing conclusions about ecotypes based on short‐term studies, given that contemporary genetic differentiation, although low, might indicate speciation in progress (e.g. Chiesa et al., 2014; Menz et al., 2015). Notwithstanding rapid advances in genetic methods and resources for nonmodel organisms (Hunter et al., 2018; Segelbacher et al., 2021), the lack of available genomic resources for most such taxa have so far limited prospects for investigating the possible existence of adaptive genetic differentiation among populations that differ in phenotype, diet and habitat use (Crandall et al., 2000; Waples, 1995). Additionally, detecting population‐level structure in continuously distributed species can be difficult, as genetic structure can be characterized by clinal patterns across broad regions rather than discrete clusters (Priadka et al., 2019). Moreover, highly connected populations with large effective population size (N_e_) often show very weak genetic differentiation, which reduces the power of genetic tools to assign individuals to the population of origin (Asaduzzaman et al., 2019). Spatiotemporal fluctuations in gene flow can also confound interpretation and require resolution by means of genome‐wide profiles that allow investigation of adaptive variation (e.g. Le Moan et al., 2016). More species have had their genomes sequenced in recent years, increasing the number of available genetic markers and promoting new insights into evolutionary history and adaptive processes (e.g. Foote et al., 2015, 2016; Taylor et al., 2019, 2020), whereas taxa such as amphibians and reptiles have comparatively few available resources (Fuentes‐Pardo & Ruzzante, 2017). Without a reference genome for the target species, it is possible to use that of a related species, although this requires considerable caution for study design and data analyses (Bentley & Armstrong, 2021; Fuentes‐Pardo & Ruzzante, 2017). We posit that these diverse stages of development might help explain the problems in applying a consistent concept of ecotype for evolutionary biology and conservation management.

A consistent and practical ecotype concept matters for local management and for broad‐scale and international conservation efforts. Incorporating genetic measures that consider existing variability (quantifiable differentiation from other groups) and relevant dimensions of future evolutionary potential (Barbosa et al., 2018; Hoban et al., 2020; Milot et al., 2020) has consequences for conservation planning. Evolutionary change is a continuous natural process that produces and sustains biodiversity (Sgrò et al., 2011). Although new methods and data permit increasingly higher resolution, the delineation of taxonomic boundaries will inherently involve certain grey areas, given that evolution is an ongoing process where making objective decisions about whether two populations belong to the same species can be difficult (Stanton et al., 2019; Zachos, 2018). Adaptations to distinct natural conditions (e.g. high altitudes; Cheviron & Brumfield, 2012) or human‐induced environments (e.g. disturbance; Gaynor et al., 2018) can also be relevant for environmental planning, where recognizing conservation units below the species or subspecies such as distinct population segments (Haig et al., 2006) or ecotypes could be vital (e.g. Hendricks et al., 2019; Klütsch et al., 2016). The designation of species and conservation units, and by which method(s), has been the subject of extensive deliberation (e.g. Camargo & Sites, 2013; Carstens et al., 2013; Crandall et al., 2000; Ford, 2004; Leaché et al., 2014; Luo et al., 2018; Waples, 1995, 2006). Importantly, genomic methods can be used to investigate and characterize adaptive potential independent of any particular species concept (Stanton et al., 2019), and the ecotype concept can offer clear benefits in this regard (Box 1 Part B). Genome‐wide data for nonmodel organisms are increasingly important in research and management. Depending on their evolutionary trajectory and new findings, some ecotypes might in the future be elevated to higher taxonomic ranks, as discussed for killer whales (*Orcinus orca*, Moura et al., 2015; Whitehead, 2017). Better recognition of local ecological relationships, and the realization that adaptation to rapid climate change is the only way for some wild organisms to persist (e.g. Riquet et al., 2019), can also promote more evolutionarily enlightened management (Ashley et al., 2003) and inform priorities for conservation planning.

The conservation utility of designating taxonomic units below the species levels has long been under discussion (e.g. Phillimore & Owens, 2006). Such discussions include the process of listing groups or populations at risk and allocating resources to their recovery (Haig et al., 2006; Note S1). Species are often given a higher degree of protection than that afforded to lower‐level units such as subspecies and populations (Berta & Churchill, 2012), although there are exceptions such as range‐edge species (COSEWIC, 2015; Green, 2005). Variable definitions of evolutionary potential and whether to focus on biodiversity patterns or biodiversity‐generating processes can lead to different priorities and prescriptions for conservation where some forms, for example, hybrids, may be inconsistent with conservation values (Milot et al., 2020).

The aim of this study is to show how a more consistent identification of ecotypes can advance evolutionary research and applied conservation, whereby ecotypes may be afforded more recognition as units that merit protection by representing vital intraspecific biodiversity and evolutionary potential. To achieve this aim, we first illustrate how substantial variation in the definition of ecotypes has limited consistent use. Subsequently, we suggest improved integration of ecological inference with genetic variation. Here, we show how new genomic methods permit greater awareness of genetic structure—including spatiotemporal distribution of functional and neutral genetic variation—to identify (or confirm) ecotypes and advance study of environmental factors producing and maintaining local adaptations. Specifically, we (1) review publications from a Web of Science search to determine the present characteristics used to define ecotypes in the context of conservation and management, and (2) discuss factors that could limit or confound integration of the ecotype concept with analyses in landscape, evolutionary and conservation genomics. Finally, we (3) offer recommendations for investigating and integrating genetic structure relevant to ecotypes, including problematic groups such as natural hybrid organisms and putative anthropogenic or urban ecotypes, where parallel selective pressures such as tolerance to higher temperatures may promote important adaptations to global climate change. By expanding the focus from specific genes or genome regions towards environmental processes and variables (e.g. use of marine resources by terrestrial wildlife; Box 2), we also discuss how researchers and managers can examine environmental selection across multiple species to advance broad‐scale conservation planning.

BOX 2 **The influence of marine resources on pacific coastal wolves and bears**

Wolf (*Canis lupus*) populations in the outer regions of the Pacific Coast of North America have a closer ecological relationship with salmon and other marine resources than their conspecifics farther inland, although populations occur well within dispersal distance (Muñoz‐Fuentes et al., 2009; Stronen et al., 2014). The increasing recognition of Pacific Coastal wolves as a distinct ecotype (Hendricks et al., 2019; Schweizer et al., 2016), also evident by the marine‐sourced isotopes in their diet (Darimont et al., 2009) and the presence of gastrointestinal parasites obtained from fish (Bryan et al., 2012), is therefore important for broad‐scale conservation planning in the region. Coastal bear ecology is also closely linked to salmon (Hilderbrand et al., 1999) and this resource is important for grizzly bears (*Ursus arctos horribilis*) and black bears (*U*. *americanus*). The latter comprises the local white form known as the Kermode or Spirit bear, a recessive homozygote morph where a recent study found the relevant allele occurring in a broader area, but with a lower landscape‐level frequency (up to 26%), than had previously been estimated (Service et al., 2020). Isotopic analyses of both forms show significantly higher use of marine resources in the white morph, suggesting that ecological differentiation helps maintain this polymorphism, potentially linked to increased fishing success in white bears (Reimchen & Klinka, 2017). The seasonal influence of marine resources is also evident in a broader range of taxa within this region, including mammals (Ben‐David et al., 1997), invertebrates and birds (Christie et al., 2008). Ecological data can be drawn upon more extensively for designing genetic studies that seek to illuminate evolutionary processes across taxa, for instance, the influence of marine food sources for terrestrial species occupying coastal areas (Hilderbrand et al., 1999; Muñoz‐Fuentes et al., 2009). The importance of marine food sources can thus be taken into consideration for regional conservation management of the broader ecosystem, and future genomic research could help clarify whether local bear species and other taxa exhibit parallel genetic structure to that observed in wolves.

Box 2 Images. Coastal wolves (photo: Klaus Pommerenke) and a Kermode or Spirit bear capturing salmon (photo: Paul C. Paquet).

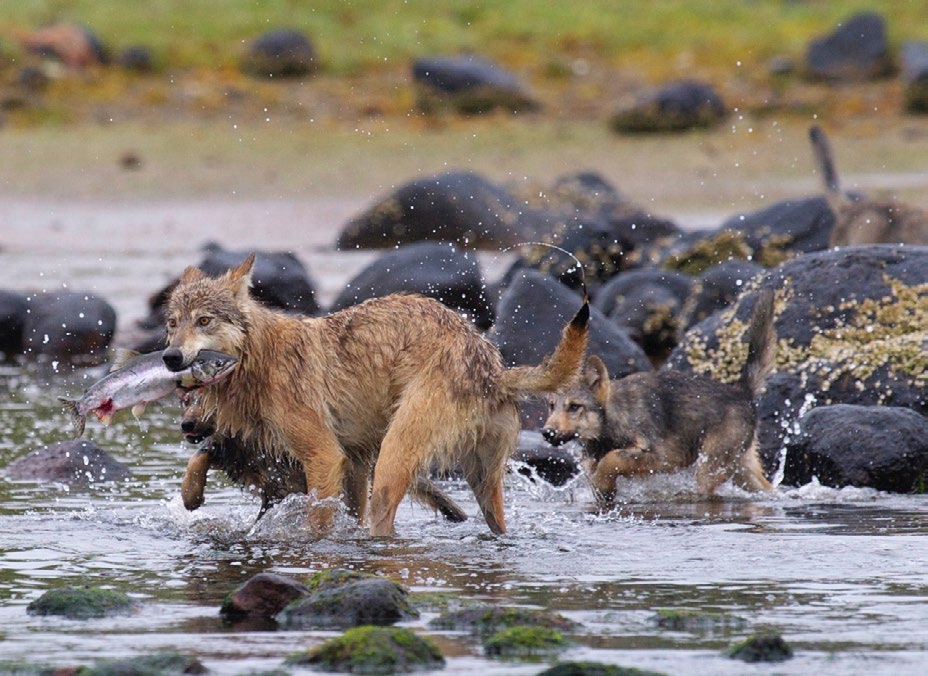

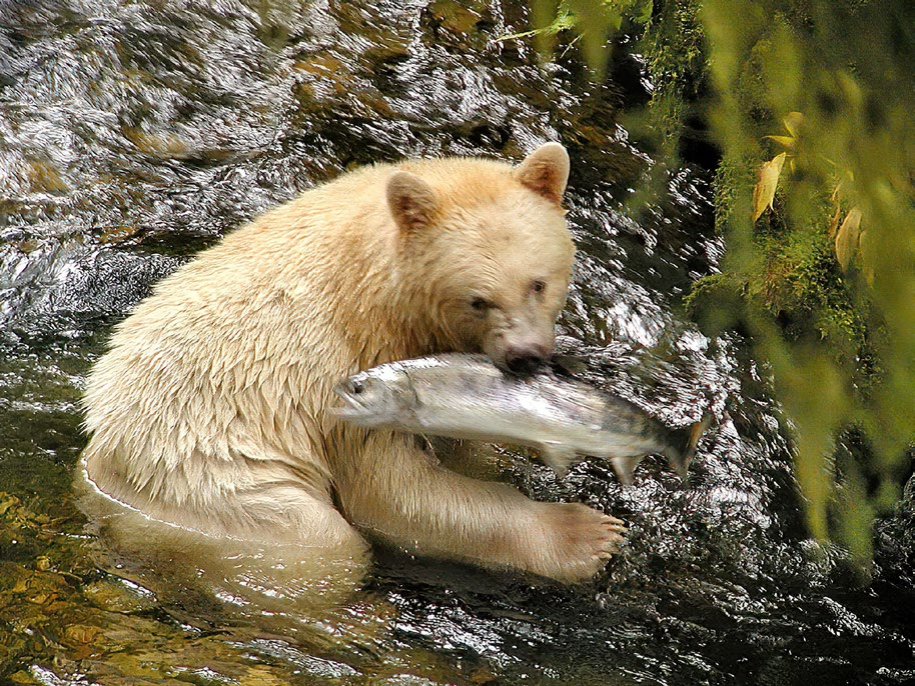

## 2 LITERATURE REVIEW

**FIGURE 1:**
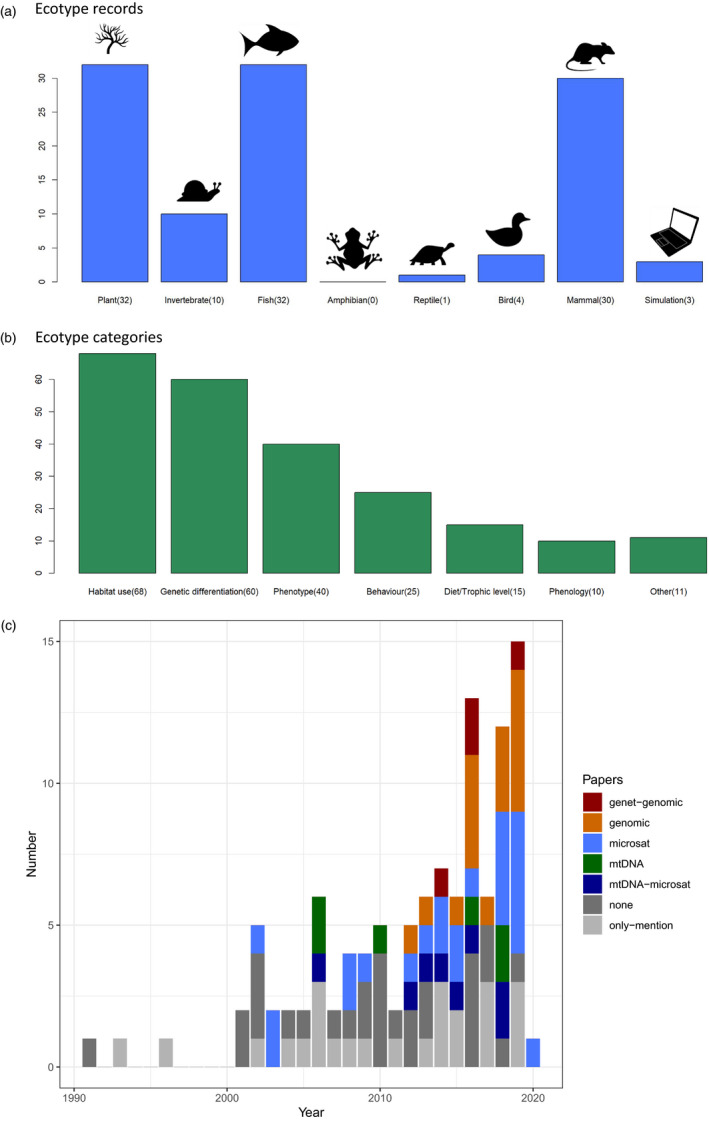
(a) Ecotype records per taxonomic group in 112 publications obtained from a search for *ecotype*, *conservation* and *management*, including three simulation and review articles. Plants, fish and mammals dominate the findings, although this is also likely to reflect the attention and resources given to these groups. (b) Ecotype categories recorded in 112 publications obtained from a search for *ecotype*, *conservation* and *management*. We noted all categories listed in each publication. The ‘other’ included pollinator species and anthropogenic ecotypes (plants), production (birds [indigenous African chicken]), parasite fauna, predation level and differences in maturation times (fish), isotope (mammal) and life history (fish and mammals). (c) Genetic resources used for 112 publications obtained from a search for *ecotype*, *conservation* and *management*. Our literature search ended in mid‐January 2020, and thus only one record is included for this year. Categories reported are as follows: ‘only‐mention’ (authors mentioned, but did not use genetic or genomic analyses); ‘none’ (no mention of genetic analyses); ‘mtDNA‐microsat’ (included mitochondrial DNA and microsatellite analyses); ‘mtDNA’ (mtDNA analyses); ‘microsat’ (microsatellite analyses); ‘genomic’ (genomic analyses); ‘genet‐genom’ genetic (mtDNA or microsatellite) and genomic analyses. Records with genomic analyses included 20 studies that reported use of SNPs, of which six used RAD‐seq, one employed transcriptome analyses and two used whole‐genome sequencing

We performed a literature search in Web of Science (https://www.webofscience.com/wos/woscc/basic‐search) that included records published until 23 January 2020 with the terms ‘ecotype AND conservation AND management’ in the topic field, which searches title, abstract and keywords. Although our criteria thereby narrowed the focus of the literature search, it facilitated our central aim of evaluating how consistently the ecotype concept has been used in applied research directly relevant to conservation and management. Moreover, our search was done without bias towards specific taxonomic groups or organisms with more available genomic resources. We obtained *n* = 118 records. Further reading revealed six records where ‘ecotype’ referred to features or entities not directly applicable for genetic analyses (e.g. habitat types such as sand dunes). We excluded these records but retained three simulation/review studies, resulting in *n* = 112 publications (one study encompassed invertebrates and plants; hence, there are 113 records). We divided taxa into seven groups (Figure 1a) and examined author classification of ecotype into the broad categories of behaviour, diet/trophic level, genetic differentiation, habitat use, phenology, phenotype and ‘other’, noting all characteristics reported in the reviewed studies (Figure 1b, Table S1). Hence, in cases where ecotype appeared to have been classified as both behaviour and habitat use by an organism (such as a fish exhibiting choices of spawning habitats), we listed both categories. We categorized genetic and/or genomic methods used in the 112 publications and noted papers that mentioned genetic/genomic analyses even if these methods were not used in the study (Figure 1c). Among 20 publications that included genomic analyses, 2 concerned birds (domesticated) and the other 18 involved fish (Table S1).

The highest number of ecotype records linked to genetic differentiation was found in fish (Table S1). This might be explained by recent efforts to develop high‐resolution genomic markers for fish (Le Moan et al., 2016), possibly owing to increasing concerns around hybridization and/or the economic importance of these taxa (e.g. Veale & Russello, 2016). In contrast, mammal ecotypes were most often reported in connection with habitat use which, in turn, may be more easily documented for terrestrial than aquatic species. Of the mammal records, 33% (10 of 30) were studies of caribou (*Rangifer tarandus*), and only two of these listed ecotypes linked to genetic differentiation, whereas seven reported habitat use. Caribou are a well‐known species of conservation concern for which there have been different and sometimes confounding results on the relationship between genetic structure and ecotypes (e.g. Courtois et al., 2003, Klütsch et al., 2016; Yannic et al., 2016, 2017; Taylor et al., 2020, Figure S1, Note S2).

Our review showed highly uneven representation among taxa, with fish, mammals and plants being subject to more ecotype research (as defined by our search criteria), and fish ecotypes most frequently being defined by genetic differentiation, whereas mammals were more often categorized by habitat use. The ecotype concept may thus have appeared more relevant for certain taxa in the context of conservation and management. In contrast, we found substantially fewer records for invertebrates, birds and reptiles, and none for amphibians. The variability might indicate that less ecotype‐related research has been done within certain fields, or that one or more of the search terms were not included, and the use of alternate names for conservation units (e.g. Ford, 2004) rather than ‘ecotype’ was preferred. Although speculative, differences among taxa might also in part reflect the training of individual scientists, and the different approaches and methods of researchers trained in ecology and genetics. To conceptualize ecotype, the habitats specific animals use need to be considered, especially for events critical to genetic exchange. When organisms choose specific localities for reproduction (spawning and calving), the resulting ecotypes can be classified in terms of behaviour and/or habitat. If fewer biologists with background in behaviour have investigated ecotype‐related questions, this could have influenced our findings. From the studies that reported ecotypes based on nongenetic categories, whether genetic differences had been considered relevant for investigation was not always apparent. Conversely, it is possible that increased availability in genetic resources for certain taxa has resulted in more geneticists taking an interest in investigating ecotypes, especially where ecotypes have been previously defined based on observable differentiation in morphology or habitat use. Furthermore, much research is focused on economically important organisms (Pauls et al., 2013). Therefore, the variability in reports of ecotypes based on genetic differentiation might primarily reflect where biologists have been able to do detailed investigation.

## 3 CHALLENGES FOR INTEGRATING GENETIC ANALYSES WITH ECOTYPES

### 3.1 Units for evolutionary research and conservation management

From our literature review, it remains unclear whether ecotypes can occur without genetic differentiation, as several studies did not include or mention analyses of genetic structure (Table S1). Examples with immediate relevance for conservation management are the Designatable Units available for assessment by the Committee on the Status of Endangered Wildlife in Canada (COSEWIC) (COSEWIC, 2011; Green, 2005). Designatable units are recognized as significant and discrete entities, where a discrete population or group of populations can be determined by one or more of three criteria comprising genetic differentiation, natural disjunction between ranges and occupancy of different ecoregions (https://cosewic.ca/index.php/en‐ca/reports/preparing‐status‐reports/guidelines‐recognizing‐designatable‐units). These guidelines recognize evolutionary significance as phylogenetic divergence, ecological (and likely adaptive) differentiation, a remnant population representing a natural occurrence, or a population whose loss would cause extensive range disjunction. The United States Endangered Species Act also permits assignment of Distinct Population Segments for vertebrate populations (reviewed in Waples, 2006) that are described geographically instead of biologically (https://www.fws.gov/pacific/news/grizzly/esafacts.htm). Although genetic data are typically included, such groupings could thus encompass units that are linked to specific habitat types without considerations of genetic criteria, and this uncertainty complicates the delineation and monitoring of evolutionary and conservation units, including ecotypes. Genetic structure plays an important role in defining management units for wild species, including endangered (Collins et al., 2017) and invasive (Haynes et al., 2009) species. Turesson (1922) underlined the importance of heritable differences as central to the ecotype concept. If ecotypes are reported based on phenotype, behaviour or other criteria without mention of genetic differentiation, such units may instead reflect phenotypic plasticity and the ability to exploit spatiotemporal differences in available resources. Although research on species such as killer whales suggests the relationship between plastic and heritable trait in ecological speciation merit further long‐term study (Foote, 2012), plastic traits may be of limited value in distinguishing evolutionary differences relevant for conservation management (Box 1 Part B). Here, preserving genome‐wide variability may have greater priority as insurance aimed at retaining long‐term evolutionary potential (Kardos et al., 2021), which can be defined according to the organism and conservation context under consideration (Milot et al., 2020).

The ecotype concept serves to focus on the essential relationship between organisms and their environment (Morrison, 2012), and we highlight this aspect by comparing definitions for units relevant for evolutionary research and conservation management (Table 1). Although not intended as a comprehensive list, Table 1 shows that (i) ecotype units specifically address ecological distinctness, whereas other concepts appear less explicit about making such assumptions. Different concepts also seem to vary in the extent to which they balance adaptation (including ecological distinctness) and isolation (Waples & Lindley, 2018). Moreover, Table 1 illustrates that (ii) ecotype definitions are inconsistent in the degree to which they require adaptive genetic variation. We support the ecotype definition by Le Moan et al. (2016): ‘populations of the same species which have evolved heritable physiological, morphological, behavioural or life history differences that are closely associated with environmental variation’, which specifies adaptive genetic variation while encompassing broad ecotype categories relevant across taxa. Although implicit in the Le Moan et al. (2016) definition, we highlight the importance of, and encourage the use of, adaptive genetic variation as an explicit consideration in the ecotype concept. Future research (e.g. on polygenic traits and epigenetics) might revisit and refine how ecotypes are categorized. However, the ecotype concept can aid identification and monitoring of locally adapted populations and, where needed, their designation for protection, independent of (or possibly in parallel with) taxonomic classifications. Given rapid environmental changes and the urgent need to conserve genetic diversity and adaptive capacity across taxa at a global scale (Hoban et al., 2020), ecotypes thus offer a concrete yet flexible framework for investigating and conserving evolutionary potential.

**TABLE 1:** Definition of concepts to describe units relevant for conservation management and evolutionary research. The list is not intended to be exhaustive, but to illustrate how ‘ecotype’ relates to other concepts and some of the similarities and discrepancies used in defining ecotypes in the literature. Below we attribute definitions to key publications and highlight taxa used as examples in the papers. We highlight the importance of ecological discreteness and adaptive genetic variation in the definition of various concepts, and whether these conditions appear focal, included, possible or not obvious. A proposed ecotype definition is highlighted in bold font

Concept	Definition	Reference	Taxonomic unit(s) addressed	Ecological discreteness/Adaptive genetic variation
Subspecies	‘Populations partway through the evolutionary process of divergence towards full speciation’	Frankham et al. (2002)	Across taxa	Included/Included
Evolutionary Significant Unit (ESU)	‘Populations possessing genetic attributes significant for present and future generations of the species in question’	Ryder (1986)	Across taxa, examples from mammals (Somali black rhino; *Diceros bicornis brucii*)	Included/Included
Evolutionary Significant Unit (ESU)	Populations that are ‘(1) substantially reproductively isolated from other conspecific population units, and (2) represent an important component in the evolutionary legacy of the species’	Waples (1991)	Fish (Pacific salmon, *Oncorhynchu*s spp.)	Included/Included
Evolutionary Significant Unit (ESU)	‘Clusters of organisms that are evolutionarily distinct and hence merit separate protection’	Vogler and Desalle (1994)	Across taxa, examples from insects (Tiger beetles, *Cicindela* spp.)	Possible/Not obvious
Evolutionary Significant Unit (ESU)	Units that are ‘reciprocally monophyletic for mtDNA alleles and show significant divergence of allele frequencies at nuclear loci’	Moritz (1994)	Across taxa	Possible/Possible
Evolutionary Significant Unit (ESU)	Units where ‘both genetic and ecological information should be used, with an emphasis placed on exchangeability instead of genetic distinctiveness’ for classification	Crandall et al. (2000), adapted from Templeton (1989)	Across taxa	Focal/Focal
Management Unit (MU)	‘Populations with significant divergence of allele frequencies at nuclear or mitochondrial loci, regardless of the phylogenetic distinctiveness of the alleles’	Moritz (1994)	Across taxa	Possible/Not obvious
Distinct Population Segment (DPS)	‘A population (or group of populations) will be considered "distinct" (and hence a "species") for purposes of the ESA if it represents an evolutionarily significant unit (ESU [defined above]) of the biological species’	Waples (1991)	Fish (Pacific salmon, *Oncorhynchus* spp.)	Included/Included
Designatable Unit (DU)	‘Discrete and evolutionarily significant units of the taxonomic species, where “significant” means that the unit is important to the evolutionary legacy of the species as a whole and if lost would likely not be replaced through natural dispersion’	COSEWIC (2018)	Across taxa	Included/Included
Ecotype	‘Ecological unit to cover the product arising as a result of the genotypical response of an ecospecies to a particular habitat’	Turesson (1922)	Plants (*Antirrhinum rhinanthoides*)	Focal/Focal
Ecotype	‘An intraspecific product of environmental selection arising as a result of genotypic response to a particular habitat’	Gregor and Watson (1961), citing Turesson (1922)	Across taxa	Focal/Focal
Ecotype	‘Populations within a plant species that are genetically adapted to different ecological conditions, often of soil and climate’	Frankham et al. (2002)	Across taxa	Focal/Focal
Ecotype	‘Populations of the same species that evolved different demographic and behavioral adaptations to cope with specific ecological (biotic and abiotic) constraints’	Courtois et al. (2003), adapted from Mallory and Hillis (1998)	Mammal (caribou; *Rangifer tarandus*)	Focal/Focal
Ecotype	‘Distinct genotypes (or populations) within a species, resulting from adaptation to local environmental conditions; capable of interbreeding with other ecotypes or epitypes of the same species’	Hufford and Mazer (2003)	Plants (across taxa)	Focal/Focal
Ecotype	‘All the members of a species that are fitted to survive in a particular kind of environment within the total range of the species’	Erickson and Navarrete‐Tindall (2004), citing Clausen et al. (1945)	Plants (focused on native ecotypes from the tallgrass prairie)	Focal/Possible
Ecotype	‘Conspecific groups with similar ecological adaptations regardless of genealogical relationship’	Cronin (2006)	Across taxa	Focal/Possible
Ecotype	‘Life history variant’	D’Amelio & Wilson (2008)	Fish (brook trout; *Salvelinus fontinalis*)	Focal/Possible
Ecotype	‘A population or a group of populations adapted to a particular set of environmental conditions’	COSEWIC (2011)	Mammal (caribou; *Rangifer tarandus*)	Focal/Possible
Ecotype	‘Ecologically specialized lineages’	Riesch et al. (2012)	Mammal (killer whale; *Orcinus orca*)	Focal/Possible
Ecotype	‘Groups of populations, which are distinguished by a composite of variation in many traits and allele frequencies across loci over space… formed by multiple trait adaptations to many environmental variables that covary in space’	Lowry (2012)	Across taxa	Focal/Possible
Ecotype	‘Populations [that] show strong resource specializations based on consistent prey choice within stable, matrifocal social groups (pods), together with genetic and phenotypic differentiation’	Moura et al. (2015), citing Hoelzel et al. (1998), Pitman and Ensor (2003), Hoelzel et al. (2007), and Morin et al. (2010)	Mammal (killer whale; *Orcinus orca*)	Focal/Possible
Ecotype	‘Ecologically, genetically and geographically divergent lineages that might represent cryptic species’	Bracamonte et al. (2015)	Fish (four Australian freshwater species)	Focal/Possible
Ecotype	**‘Populations of the same species which have evolved heritable physiological**, **morphological**, **behavioural or life history differences that are closely associated with environmental variation’**	Le Moan et al. (2016)	Fish (European anchovy; *Engraulis encrasicolus*)	Focal/Focal
Ecotype	‘A plant population that originated in a specific area and has genetic adaptations to its environment’	Altrichter et al. (2017)	Plants (focus on native plants and local ecotypes for ecological restoration)	Focal/Focal
Ecotype	‘The resulting genetic divisions among wolf populations may reflect observed morphologic features related to diet (e.g. dentition, skull robustness and shape), vision (e.g. for open or closed terrain), metabolism, thermal regulation in response to ambient temperature, and locomotion (e.g. for migratory or territorial behavior) suggesting these genetic partitions may define ecological units (“ecotypes”)’	Hendricks et al. (2019)	Mammal (grey wolf; *Canis lupus*)	Focal/Focal

### 3.2 Movement vs. gene flow

A major challenge for ecotype‐relevant genetic research is occasional discrepancies between spatial population genetic structure and observed wildlife movement among populations (by radio‐ or GPS‐tagged organisms). In marine and terrestrial environments, some populations, that is, genetic clusters, occur in sympatry yet exhibit distinct spatiotemporal patterns (Vander Wal et al., 2013; Yannic et al., 2016). Overlapping ranges and migration routes complicate demarcation of designatable units for species such as beluga whales (*Delphinapterus leucas*), where logistical difficulties have limited data collection during winter (COSEWIC, 2016). Furthermore, the same landscape features can produce different patterns of movement and gene flow in related species despite a shared capacity for long‐distance dispersal (Sawaya et al., 2014). Hence, investigations of species’ home ranges, movement patterns and sites of reproduction may yield different information where each piece explains but a fraction of the complete picture of how animals use the landscape or seascape. Multiple independent methods involving direct and indirect approaches, for example, trail camera records coupled with genetic analyses, might be needed to infer animal movement among populations and how often these movements translate into gene flow. As Waples (1995) noted on identification of Evolutionary Significant Units (ESU), the degree of isolation between populations must be strong enough to allow accumulation of evolutionarily important differences, but this does not imply total separation. Dispersal and gene flow can be problematic for the delineation of units relevant for evolution and conservation in space and time. Many long‐distance dispersers die before reproducing in their new habitat (Bartoń et al., 2019), while some observed genetic structures are only transitory (Stanton et al., 2019). Of particular relevance to ecotypes are recent results demonstrating adaptive genetic differentiation despite limited or no neutral genetic structure (Asaduzzaman et al., 2019; Lemay & Russello, 2015), which can help clarify the relationship between movement and gene flow, especially in habitats experiencing rapid environmental changes.

## 4 A FRAMEWORK AND RECOMMENDATIONS FOR INCORPORATING GENETIC STRUCTURE INTO ECOTYPE DESIGNATION

Only 53% (60 of 113) of the studies we reviewed included analyses of genetic structure in their categorization of ecotypes. Therefore, asking if genetic measures were not considered necessary or informative seems relevant. Alternatively, few or no genetic markers may have been available for the organism of interest. Our temporal analysis of studies that included genetic and genomic analyses suggests that this factor could have played a role, especially for nonmodel species (Figure 1c, Table S1). We use three central questions (Box 1 Part B) to propose a framework for how future research can evaluate and incorporate adaptive genetic structure into ecotype designations, and below we discuss some considerations for each one. With this framework, we are looking for (A) the existence of ecologically relevant traits, (B) whether such traits are persistent and consistently associated with environmental variation (as opposed to indicating phenotypic plasticity) and (C) whether organisms exhibit adaptive genetic variation associated with these traits. We have used the example of the killer whale to illustrate the process, which is summarized below in C after addressing some considerations for the first two steps.

### 4.1 Identification and conservation of ecologically relevant traits

#### 4.1.1 Recognizing intraspecific variation

The listing of subspecies or distinct population segments can help focus management on vulnerable areas, allowing protection of vital evolutionary potential while reducing impacts on landowners or other special interests relative to that of listing the entire species across its range (Haig et al., 2006). Growing recognition of the importance of conserving genetic diversity and adaptive potential also within species (Hoban et al., 2020) has implications for conservation legislation, and conservation can benefit by going beyond taxonomic entities and increase attention on the underlying biodiversity. One example is the ESU approach, which offers a more in‐depth measure of diversity for applied conservation (Fraser & Bernatchez, 2001; Yannic et al., 2016; Zachos, 2018). This approach would require international conservation organizations such as the International Union for Conservation of Nature (IUCN) to expand focus on units of conservation that are intraspecific and delineated in a flexible manner, with the goal of preserving the maximum amount of variability (Zachos, 2018). Ecotypes could therefore offer consequential entities for more local and flexible conservation management. Adaptive genetic differentiation and ecotypes associated with resource use, habitat and life history can now be detected based on techniques such as genotyping‐by‐sequencing and restriction site‐associated DNA sequencing or RAD‐seq (Barbosa et al., 2018; Funk et al., 2012; Nichols et al., 2016; Perreault‐Payette et al., 2017), which has also enabled accurate species delimitations based on relatively few (~100) loci (Leaché et al., 2014). Even so, these and other studies also show that key problems remain (Note S3), including detection of complex polygenic traits, where identification may be affected by the reduced genome representation of the RAD‐seq approach (reviewed in Fuentes‐Pardo & Ruzzante, 2017). RAD‐seq and other genotyping‐by‐sequencing methods do not require the presence of a sequenced genome, and they have provided rapid advances for research on nonmodel organisms (Ekblom & Galindo, 2011). Finally, observations of similar environmental adaptations in different areas indicate independent parallel evolution in separate habitats (Waples, 2006; Winchell et al., 2020) and in various taxonomic groups (e.g. Cheviron & Brumfield, 2012; Johnson & Munshi‐South, 2017; Kang et al., 2017; Taylor et al., 2020). Future research across species could help clarify how often parallel adaptations to rapid habitat and climate change occur by selection on different genes involved (e.g. in temperature tolerance), and the importance of specific genes of large effect for evolution and conservation (Waples & Lindley, 2018).

#### 4.1.2 The complexity of gene flow and gene‐targeted conservation

Identification of genomic regions under selection is the basis for understanding local adaptation and the relevant ecological factors involved (Funk et al., 2012; Kardos & Shafer, 2018), and many conservation programmes aim to preserve genetic diversity by ensuring gene flow. However, spatially restricted phenotypic variants may have unique morphology, life history traits or habitat use that signal local adaptation to ecological conditions (Crandall et al., 2000, Box 2). These rare variants might represent a unique evolutionary lineage and warrant special protection as part of an ESU (Crandall et al., 2000). An informative example is the Kermode or Spirit bear, a white‐coated colour morph of the black bear (*Ursus americanus*) found on the north Pacific Coast of Canada. This morph is linked to a recessive mutation at the melanocortin 1 receptor gene (Mc1r), and for this reason, isolation and limited gene flow may be essential for its continued persistence (Marshall & Ritland, 2002; Service et al., 2020). Recent findings also suggest that this morph has a dietary niche with higher use of marine resources than the black colour morph, and ecological segregation may therefore play a role in preserving this polymorphism (Reimchen & Klinka, 2017). However, some researchers have questioned whether conservation efforts to target specific genes and genomic regions will provide a long‐term sustainable approach, especially given rapid environmental change where it is difficult to predict exactly which genetic variation might become important in the future (Kardos & Shafer, 2018; Stanton et al., 2019; Waples, 1995). The identification and conservation of ecologically relevant traits may thus require a broad temporal perspective, where we acknowledge that such traits could be affected by genes or genome regions not yet identified, or genes that could play an increasingly important role in the future under different environmental conditions.

### 4.2 Recognition of traits linked to environmental variation.

#### 4.2.1 Habitat and environmental variation

In various species, some populations show adaptations to their local environment, as illustrated by plants adapted to conditions of drought (Exposito‐Alonso et al., 2018), and fish (Kang et al., 2017) and mammals (Werhahn et al., 2018) adapted to life at high altitudes. For some species, habitat suitability models from parts of their range are thus not reliably transferrable to other regions, with important consequences for management (Bain et al., 2015; Denryter et al., 2017). Ecological knowledge of the species under study can provide perspectives on whether observed animal movements are common or atypical; for example, regarding sex‐biased dispersal (Støen et al., 2006), and the occurrence of seasonal movements in species that are usually nonmigratory (Musiani et al., 2007) or exhibit vagrant behaviour (Kutschera et al., 2016). Ecological data can also be combined with information on the spatial distribution of genetic variation as indicators of local adaptations (Fitzpatrick et al., 2015) or responses to ongoing environmental change (Koen et al., 2014; Kutschera et al., 2016). Similarly, an ecological genetic perspective may improve our understanding of contemporary evolution in highly mobile species now expanding their range and population size in Europe (Chapron et al., 2014; Deinet et al., 2013 and references therein) and North America (Kays et al., 2010; LaRue et al., 2012). Recolonization of historic ranges over the past few decades (Chapron et al., 2014; Deinet et al., 2013) following the ‘rewilding’ of European landscapes and range expansion of species has implications for community structure and function, and may ultimately alter the selective pressures and spatial genetic structure of sympatric species. The golden jackal (*Canis aureus*), for example, has colonized areas where it was never previously recorded and is now successfully reproducing in those ranges (Kowalczyk et al., 2020).

Global climate change will have major consequences for the conservation of genetic diversity, and shows the need to investigate both neutral and functional genetic diversity to mitigate negative impacts and inform conservation planning (Hoban et al., 2020; Pauls et al., 2013). For instance, genotypes adapted to higher temperatures and drought could increase the long‐term persistence of some wild organisms (Exposito‐Alonso et al., 2018; Sgrò et al., 2011). Our understanding of climate change effects on genetic diversity is often based on commercially valuable organisms, and we have more limited knowledge of other taxa (reviewed in Pauls et al., 2013). A broader aim of conserving diversity and adaptive potential within all species is therefore urgently needed (Hoban et al., 2020).

#### 4.2.2 Adaptation to human activities

Although ecotypes are typically associated with specific natural (as opposed to anthropogenic) habitats, rapid environmental changes in urban and other anthropogenic environments also appear relevant for ecotype identification and conservation management. Recent reports have indicated differential selective pressures in urban and nonurban environments that influence morphology, physiology and behaviour (Bury & Zając, 2020; Johnson & Munshi‐South, 2017; Puckett et al., 2020). This includes responses to increased temperatures, which are observed in urbanized environments (Campbell‐Staton et al., 2020; Johnson & Munshi‐South, 2017; Winchell et al., 2020). Crucially, increased temperatures are also occurring because of global climate change, with major consequences for global biodiversity and ecological processes (Scheffers et al., 2016). Urbanization and global climate change may therefore impose certain parallel selective pressures on wild species. Populations now experiencing rapid selection towards life in urbanized environments might thus, over time, become more tolerant to increasing temperature, drought and other broad‐scale global changes. Urbanization may drive evolutionary patterns that are highly repeatable and encompass genotypes, gene regulations and phenotypes (Campbell‐Staton et al., 2020; Winchell et al., 2020). Such populations (possibly incipient ecotypes) can offer valuable models for genomic research. Urban lizards (Lacertilia), for instance, were found to tolerate higher temperatures than their conspecifics in forest environments (Campbell‐Staton et al., 2020). They found a linkage between polymorphism at the RARS protein synthesis gene and heat tolerance plasticity within ‘heat islands’ in urban environments, and signs of parallel selection among independently colonized urban environments. Conversely, analyses across related species suggest that organisms well suited to hot and dry conditions could more easily adapt to urban environments, thus offering a means of predicting future vulnerability and conservation needs (Winchell et al., 2020).

Urban environments and human‐derived food sources appear to have altered the ecology and morphology across taxa (Johnson & Munshi‐South, 2017). Such changes have also been reported in abundant species such as brown rats (*Rattus norvegicus*) with a long history in human settlements (Puckett et al., 2020). Mammals in urban and other disturbed environments were found to have lower N_e_ and genetic diversity, which may limit their ability to adapt to environmental changes (Schmidt et al., 2020), and bottleneck events and small sample sizes in urban populations can increase the risk of false signals of selection (Garroway & Schmidt, 2020). Emerging findings on the speed and direction of selection from species with well‐known genomes, including the extent to which observed changes are due to phenotypic plasticity (Campbell‐Staton et al., 2020; Johnson & Munshi‐South, 2017; Pauls et al., 2013; Puckett et al., 2020), could inform investigations of contemporary evolution in lesser known organisms and subsequent conservation planning in areas experiencing rapid anthropogenic change.

#### 4.2.3 Social structure and ecological niche

Genetic data can be incorporated with ecological information such as stable isotope analyses, and the geographical scale of habitat use and movements, to improve the designation of meaningful conservation units (Nykänen et al., 2019; Thiemann et al., 2008). This approach can also help resolve cases where genetic units are not socially segregated but comprise individuals with dietary and behavioural differences, to show whether a process of niche specialization and emergence of ecotypes might have started and require further study (Tavares et al., 2018). Investigations into ecologically significant genetic structure may be especially problematic for species that occur at low densities with limited N_e_, as this parameter is considered vital for evolutionary resilience (Sgrò et al., 2011) but can be hard to determine. New genetic and genomic methods offer exciting opportunities for detecting increasingly fine‐scale genetic differences in space and time. However, we must also be aware that as analytical power increases, we augment magnification until at last we observe highly localized structures (Carstens et al., 2013), which can include basic social and family structure in wild species. Yet, some populations with high rates of mixing (those that exhibit fission–fusion) may lack these intuitive social structures (Tavares et al., 2018). In such cases, distinguishing the relevant scale at which to interpret genetic data becomes more difficult, requiring detailed ecological knowledge of the species and the population under study.

### 4.3 Application of the ecotype concept to conserve locally adapted populations

Investigating relationships among candidate genes for adaptation, their geographic distribution and environmental importance often requires broad‐scale research that is difficult and costly (Johnson & Munshi‐South, 2017; Pauls et al., 2013). Furthermore, some studies have found polygenic patterns of selection (Campbell‐Staton et al., 2020; Exposito‐Alonso et al., 2018), which are likely to be important (Cheviron & Brumfield, 2012) but often difficult to investigate (Kardos & Shafer, 2018; Pritchard & Di Rienzo, 2010). In‐depth study of biochemical pathways and gene expression are also likely to clarify the paths from genotype to phenotype for environmental adaptations (Cheviron & Brumfield, 2012). As an example of the proposed framework (Box 1 Part B), we use the killer whale, where recent research, combining new genomic methods with findings from ecological research on diet, behaviour and habitat use, illustrates categorization of ecotypes. Here, we illustrate how new genomic analyses have permitted (A) identification of ecologically relevant traits that are (B) persistent and affiliated with environmental variation, and where organisms (C) exhibit associated genetic variation known (or expected) to be adaptive.

Although certain killer whale ecotypes are relatively well known, additional information is needed for conservation management, especially for populations that are logistically more difficult to study (de Bruyn et al., 2013; COSEWIC, 2008; Lefort et al., 2020; Riesch et al., 2012). To date, extensive research has nonetheless demonstrated the existence of genetically divergent units that differ in behaviour, diet, phenotype, group size and vocalization patterns (e.g. Foote et al., 2016; Hoelzel et al., 2007; Moura et al., 2015; Riesch et al., 2012). This includes ‘resident’ killer whales on the Pacific Coast of Canada that eat mainly fish, whereas partially sympatric ‘transient’ killer whales feed largely on marine mammals (COSEWIC, 2008). Transients have been reported to be larger in size, but to form smaller groups that are typically less vocal (reviewed in Foote, 2012). These characteristics thus provide an affirmative answer to question A (Box 1 Part B) about the existence of different traits. Furthermore, these traits appear to be highly persistent and consistent with environmental variation (question B); ecological and genetic research have shown that several groups are clearly segregated despite high mobility, partial range overlap and a lack of physical barriers to dispersal (COSEWIC, 2008; Foote et al., 2016), and phylogenetic analyses have indicated long‐term isolation in several ecotypes on the scale of thousands of years (Foote et al., 2016; Hoelzel et al., 2007; Morin et al., 2010; Riesch et al., 2012). In Canada, COSEWIC has designated five DUs for conservation management of the species based on various combinations of morphology, genetic differentiation, geographic range, plus social, foraging and acoustic behaviour (COSEWIC, 2008). Some populations thus have overlapping range but different behaviour and diet, whereas others have disjunct ranges but may have more similar diets (COSEWIC, 2008). The framework's final query asks for adaptive (functional) variation linked to persistent (heritable) traits associated with environmental variation (question C), a topic where genomic resources have been instrumental in advancing new knowledge. Genomic analyses of killer whale ecotypes have reported adaptive variation associated with several traits linked to environmental variation, including cold adaptation, dietary variation and reproductive function (Foote et al., 2016). Forthcoming research combining genomics, satellite tracking, dietary analyses and other data can offer further resolution on killer whale evolutionary history (Whitehead, 2017), including ecotypes that have so far proved more difficult to study (COSEWIC, 2008; Lefort et al., 2020) and populations where patterns of possible ecological divergence are currently less clear (de Bruyn et al., 2013; Tavares et al., 2018). Killer whale ecotypes may now represent incipient (sub)species (Morin et al., 2010; Moura et al., 2015; Whitehead, 2017). They could have originated as small groups of maternally related individuals where, over time, an initial plastic behavioural response (question B) augmented by social learning influenced natural selection and ecotype development (Foote et al., 2016). Hence, the cultural transfer of knowledge could hasten the development of ecotypes (Riesch et al., 2012) but might also constrain adaptation to rapidly changing environmental conditions, with consequences for their long‐term conservation status and management (COSEWIC, 2008). Some potential killer whale ecotypes may be more difficult to categorize depending on their stage in the developmental process (Riesch et al., 2012; Tavares et al., 2018). Given considerable variation in life and demographic histories across species, the development of (incipient) ecotypes in this and other taxa could therefore occur at different spatiotemporal scales.

#### 4.3.1 Parallel ecotypes and polygenic effects

Certain adaptations appear to have evolved in parallel in response to independent selection in similar environments (e.g. Campbell‐Staton et al., 2020; Rennison et al., 2019; Taylor et al., 2020). In theory, this might represent either one or multiple ecotypes. Possibly, previous gradual environmental changes permitted evolution of traits involving many variants with small fitness effects, which could have facilitated parallel evolution, but that are often difficult to detect with standard statistical approaches (Burns & Novikova, 2020). However, the situation may be different for the rapid changes occurring at present, where standing variation provides important raw material for selection, but the speed of change exceeds the possibility of adaptation even with the aid of novel variation (Burns & Novikova, 2020).

#### 4.3.2 Adaptive differentiation without neutral genetic structure

For some species, genetic differentiation was found for putatively adaptive loci and supported the delineation of ecotypes, despite a lack of obvious population structure at neutral loci (Asaduzzaman et al., 2019; Lemay & Russello, 2015). For example, recent research on fish has showed divergent local adaptations to various habitats, which has resulted in different ecotypes despite the general absence of barriers to dispersal, although the reported ecotypes were also supported by morphological and behavioural phenotypes (Asaduzzaman et al., 2019). Sinclair‐Waters et al. (2018) submit that structural reorganizations like chromosomal inversions may contribute to adaptive differences among populations and to the long‐term persistence and spatiotemporal coexistence of different ecotypes. These results also illustrate that previously confounding or inconclusive findings from neutral genetic markers can now be re‐examined with genome‐wide profiles to clarify evolutionary history, the distribution of functional genetic variation and its implications for conservation (Asaduzzaman et al., 2019).

#### 4.3.3 Emerging ecotypes

Rapid environmental modifications linked to human‐induced climate change (Scheffers et al., 2016; Urban, 2015) suggest that the ecotype concept is increasingly relevant for conservation management under altered environmental conditions, which include globally shifting patterns in precipitation and vegetation (Scheffers et al., 2016). Examples are complex groups such as instances of wild species forming hybrid ecotypes (Gante et al., 2015; Kays & Monzón, 2017; Nolte et al., 2005). Such ecotypes seem more likely to emerge where current selection regimes favour novel genetic variation obtained via introgression, and they may be linked to human habitat modifications that have facilitated range expansion (Gante et al., 2015; Guo, 2014; Monzón et al., 2014; Nolte et al., 2005). Additionally, human‐induced changes can produce novel niches where hybrid organisms may be better adapted than parental taxa (Guo, 2014; Nolte et al., 2005). Hybrid organisms resulting from natural hybridization in wild taxa merit protection but can nevertheless pose challenges for conservation management (Allendorf et al., 2001). The ecotype concept allows classification of such groups centred on their local ecological function. Despite their unresolved taxonomy (Gante et al., 2015), and although they may be rare, natural hybrid ecotypes can be recognized in conservation management of, for example, predator–prey relationships (Kays & Monzón, 2017). Rapid environmental change, to which hybrids may adapt more swiftly than parental taxa (Stanton et al., 2019; Stelkens et al., 2014), combined with expanding genomic resolution allowing their detection could make such groups increasingly common in the future.

## 5 CONCLUSIONS

Based on this study, we observed the following problems with the ecotype concept and its use: (1) at present, the role of spatial genetic structure in delineating ecotypes ranges from all important to unspecified, and this lack of consistent definition limits broader application, and (2) current discrepancies between genetic structure and ecotype may reflect lack of genomic resources for many taxonomic groups and therefore limited investigation of adaptive vs. neutral differentiation. Although existing conservation units variously include ecological distinctness and the presence of adaptive genetic diversity, the application of an ecotype concept centred on adaptive diversity can offer additional direction. Preservation of genome‐wide variation is critical for evolution and conservation (Kardos et al., 2021), and provides raw material for responses to rapid environmental changes that may be difficult to predict. By emphasizing genetic variation relevant for organisms and their relationship with their environment, the ecotype concept offers a framework for recognizing and monitoring known or putative adaptive variation in space and time. The ecotype designation can also incorporate complex entities such as hybrid organisms, emerging ecotypes and populations units that show adaptive—but little or no neutral—genetic differentiation. These insights can complement genome‐wide measures of diversity and facilitate proactive conservation planning. For example, by informing timely conservation actions such as biobanking and genetic rescue (Segelbacher et al., 2021). We suggest a framework for resolution that capitalizes on the rapidly increasing knowledge of adaptive genetic variation and relates this diversity to persistent phenotypic differences associated with environmental variation. Notably, ecological data on characteristics such as social structure, habitat use and diet can provide essential context for analyses of genetic structure by informing working hypotheses and priorities for data collection. Additionally, these ecological data can aid interpretation of findings from increasingly detailed genomic profiles and highlight important questions for management (Box 2), which can help connect evolutionary researchers with practitioners in ecology and conservation. Genome‐wide profiles offer unparalleled opportunities to learn how selective pressures have shaped wild species and their ecological functions, thereby helping us to identify ecotypes, their spatial distribution and the processes most important in driving evolutionary change.

## CONFLICT OF INTEREST

The authors declare no conflict of interest.

## Supporting information

Supplementary Material

## Data Availability

Data sharing is not applicable to this article as no new data were generated or analysed in this study.
