# Supplementary material for: The relevance of genetic structure in ecotype designation and conservation management
Source: Evol Appl. 2022 Jan 19;15(2):185–202. doi: 10.1111/eva.13339 (PMC8867706; doi:10.1111/eva.13339)
Supplement: Supplementary file 1 — Supplementary Material [file EVA-15-185-s001.pdf]

Supplemental materials for "The relevance of genetic structure in ecotype designation and conservation management"

Supplemental Table S1. Ecotype criteria reported across taxa in a literature review of 112 publications from a Web of Science search for "ecotype AND conservation AND management". Several studies reported multiple criteria for ecotype. One study considered plants and invertebrates, and three (simulation/review) were not specific to any taxon. No records were found for amphibians.

**Taxa (n*)**	**Behaviour**	**Diet/trophic level**	**Genetic differentiation**	**Habitat use**	**Phenology**	**Phenotype**	**Other†**	**Reference number§**
Birds (4)	0	0	3	0	0	2	1	1-4
Fish (32)	14	4	27	20	5	18	3	5-36
Invertebrates (10)	3	2	0	6	1	4	0	37-46
Mammals (30)	8	9	15	23	0	6	2	47-76
Plants (32)	n/a	0	13	15	4	9	5	45, 77-107
Reptiles (2)	0	0	1	2	0	1	0	108-109
Across taxa (3; review/simulation)	0	0	1	2	0	0	0	110-112
Total no. studies	25	15	60	68	10	40	11	112

* Number of studies reviewed, including one study that included both invertebrates and plants.

† Pollinator species and anthropogenic ecotypes (plants), production (birds [indigenous African chicken]), parasite fauna, predation level and differences in maturation times (fish), isotope (mammal), and life history (fish and mammals).

§ Genomic analyses were reported for 20 studies, two for birds (reference number 2-3) and 18 for fish (reference number 5, 8-16, 26-27, 29-31, 33-34, 36).

References

1. Pham, M.H., Berthouly-Salazar, C., Tran, X.H., Chang, W.H., Crooijmans, R.P.M.A., Lin, D.Y., … Chen, C.F. (2013). Genetic diversity of Vietnamese domestic chicken populations as decision-making support for conservation strategies. *Animal Genetics, 44*, 509-521.

2. Riztyan, Katano, T., Shimogiri, T., Kawabe, K., Okamoto, S. (2011). Genetic diversity and population structure of Indonesian native chickens based on single nucleotide polymorphism markers. *Poultry Science, 90*, 2471-2478.

3. Mwambene, P.L., Kyallo, M., Machuka, E., Githae, D., Pelle, R. (2019). Genetic diversity of 10 indigenous chicken ecotypes from Southern Highlands of Tanzania based on Major Histocompatibility Complex-linked microsatellite LEI0258 marker typing. *Poultry Science, 98*, 2734-2746.

4. Soltan, M., Farrag, S., Enab, A., Abou-Elewa, E., El-Safty, S., Abushady, A. (2018). Sinai and Norfa chicken diversity revealed by microsatellite markers. *South African Journal of Animal Science, 48*, 307-315.

5. Fitzpatrick, S.W., Gerberich, J.C., Angeloni, L.M., Bailey, L.L., Broder, E.D., Torres-Dowdall, J., … Funk, W.C. (2016). Gene flow from an adaptively divergent source causes rescue through genetic and demographic factors in two wild populations of Trinidadian guppies. *Evolutionary Applications, 9*, 879-891.

6. Bracamonte, S.E., Smith, S., Hammer, M., Pavey, S.A., Sunnucks, P., Beheregaray, L.B. (2015). Characterization of MHC class IIB for four endangered Australian freshwater fishes obtained from ecologically divergent populations. *Fish & Shellfish Immunology, 46*, 468-476.

7. Liu, D., Li, Y.Y., Tang, W.Q., Yang, J.Q., Guo, H.Y., Zhu, G.L., Li, H.H. (2014). Population structure of *Coilia nasus* in the Yangtze River revealed by insertion of short interspersed elements. *Biochemical Systematics and Ecology, 54*, 103-112.

8. Chiesa, S., Piccinini, A., Lucentini, L., Filonzi, L., Nonnis Marzano, F. (2014). Genetic data on endangered twaite shad (Clupeidae) assessed in landlocked and anadromous populations: one or more species? *Reviews in Fish Biology and Fisheries, 24*, 659-670.

9. Lemay, M.A., Russello, M.A. (2015). Genetic evidence for ecological divergence in kokanee salmon. *Molecular Ecology, 24*, 798-811.

10. Bernatchez, S., Laporte, M., Perrier, C., Sirois, P., Bernatchez, L. (2016). Investigating genomic and phenotypic parallelism between piscivorous and planktivorous lake trout (*Salvelinus namaycush*) ecotypes by means of RADseq and morphometrics analyses. *Molecular Ecology, 25*, 4773-4792.

11. Arciniega, M., Clemento, A.J., Miller, M.R., Peterson, M., Garza, J.C., Pearse, D.E. (2016). Parallel evolution of the summer steelhead ecotype in multiple populations from Oregon and Northern California. *Conservation Genetics, 17*,165-175.

12. Nichols, K.M., Kozfkay, C.C., Narum, S.R. (2016). Genomic signatures among *Oncorhynchus nerk*a ecotypes to inform conservation and management of endangered Sockeye Salmon. *Evolutionary Applications, 9*, 1285-1300.

13. Le Moan, A., Gagnaire, P.A., Bonhomme, F. (2016). Parallel genetic divergence among coastal-marine ecotype pairs of European anchovy explained by differential introgression after secondary contact. *Molecular Ecology, 25*, 3187-3202.

14. Veale, A.J., Russello, M.A. (2016). Sockeye salmon repatriation leads to population re-establishment and rapid introgression with native kokanee. *Evolutionary Applications, 9,* 1301-1311.

15. Perreault-Payette, A., Muir, A.M., Goetz, F., Perrier, C., Normandeau, E., Sirois, P., Bernatchez, L. (2017). Investigating the extent of parallelism in morphological and genomic divergence among lake trout ecotypes in Lake Superior. *Molecular Ecology*, *26*, 1477-1497.

16. Lemay, M.A., Donnelly, D.J., Russello, M.A. (2013). Transcriptome-wide comparison of sequence variation in divergent ecotypes of kokanee salmon. *BMC Genomics, 14*, 308.

17. Russello, M.A., Kirk, S.L., Frazer, K.K., Askey, P.J. (2012). Detection of outlier loci and their utility for fisheries management. *Evolutionary Applications*, *5*, 39-52.

18. Wilson, C.C., Stott, W., Miller, L., D'Amelio, S., Jennings, M.J., Cooper, A.M. (2008). Conservation genetics of Lake Superior brook trout: Issues, questions, and directions. *North American Journal of Fisheries Management, 28*, 1307-1320.

19. D'Amelio, S., Wilson, C. (2008). Genetic population structure among source populations for coaster brook trout in Nipigon Bay, Lake Superior. *Transactions of the American Fisheries Society, 137*, 1213-1228.

20. Fillatre, E.K., Etherton, P., Heath, D.D. (2003). Bimodal run distribution in a northern population of sockeye salmon (*Oncorhynchus nerka*): life history and genetic analysis on a temporal scale. *Molecular Ecology, 12*, 1793-1805.

21. Chebanov, M.S., Karnaukhov, G.I., Galich, E.V., Chmir, Y.N. (2002). Hatchery stock enhancement and conservation of sturgeon, with an emphasis on the Azov Sea populations. *Journal of Applied Ichthyology, 18*, 463-469.

22. Douglas, M.R., Brunner, P.C. (2002). Biodiversity of Central Alpine *Coregonus* (Salmoniformes): Impact of one-hundred years of management. *Ecological Applications, 12*, 154-172.

23. Largiadèr, C.R., Scholl, A. (1996). Genetic introgression between native and introduced brown trout *Salmo trutta* L populations in the Rhone River Basin. *Molecular Ecology, 5*, 417-426.

24. Nordahl, O., Koch-Schmidt., P., Sunde, J., Yildirim, Y., Tibblin, P., Forsman, A., Larsson, P. (2019). Genetic differentiation between and within ecotypes of pike (*Esox lucius*) in the Baltic Sea. *Aquatic Conservation-Marine and Freshwater Ecosystems, 29*, 1923-1935.

25. Xue, D.-X., Yang, Q.-L., Li, Y.-L., Zong, S.-B., Gao, T.-X., Liu, J.-X. (2019). Comprehensive assessment of population genetic structure of the overexploited Japanese grenadier anchovy (*Coilia nasus*): Implications for fisheries management and conservation. *Fisheries Research, 213*, 113-120.

26. Morissette, O., Sirois, P., Wilson, C.C., Laporte, M., Bernatchez, L. (2019). The role of ecotype-environment interactions in intraspecific trophic niche partitioning subsequent to stocking. *Ecological Applications, 29*, e01857.

27. Svedang, H., Barth, J.M.I., Svenson, A., Jonsson, P., Jentoft, S., Knutsen, H., Andre, C. (2019). Local cod (*Gadus morhua*) revealed by egg surveys and population genetic analysis after longstanding depletion on the Swedish Skagerrak coast. *ICES Journal of Marine Science, 76*, 418-429.

28. Roney, N.E., Oomen, R.A., Knutsen, H., Olsen, E.M., Hutchings, J.A. (2018). Fine-scale population differences in Atlantic cod reproductive success: A potential mechanism for ecological speciation in a marine fish. *Ecology and Evolution, 8*, 11634-11644.

29. Morissette, O., Sirois, P., Lester, N.P., Wilson, C.C., Bernatchez, L. (2018). Supplementation stocking of Lake Trout (*Salvelinus namaycush*) in small boreal lakes: Ecotypes influence on growth and condition. *PLoS ONE, 13(7),* e0200599.

30. Johansen, T., Westgaard, J.-I., Seliussen, B.B., Nedreaas, K., Dahle, G., Glover, K.A., Kvalsund, R., Aglen, A. (2018). "Real-time" genetic monitoring of a commercial fishery on the doorstep of an MPA reveals unique insights into the interaction between coastal and migratory forms of the Atlantic cod. *ICES Journal of Marine Science, 75,* 1093–1104.

31. Sinclair-Waters, M., Bradbury, I.R., Morris, C.J., Lien, S., Kent, M.P., Bentzen, P. (2018). Ancient chromosomal rearrangement associated with local adaptation of a postglacially colonized population of Atlantic Cod in the northwest Atlantic. *Molecular Ecology, 27,* 339-351.

32. Orio, A., Bergström, U., Casini, M., Erlandsson, M., Eschbaum, R., Hüssy, K., Lehmann, A., Ložys, L., Ustups, D., Florin, A.-B. (2017). Characterizing and predicting the distribution of Baltic Sea flounder (*Platichthys flesus*) during the spawning season. *Journal of Sea Research, 126,* 46-55.

33. Riquet, F., Lieutard-Haag, C., Serluca, G., Woodall, L., Claude, J., Louisy, P., Bierne, N. (2019). Effective population size and heterozygosity-fitness correlations in a population of the Mediterranean lagoon ecotype of long-snouted seahorse *Hippocampus guttulatus*. *Conservation Genetics, 20,* 1281-1288.

34. Asaduzzaman, M., Wahab, M.A., Rahman, M.J., Nahiduzzzaman, M., Dickson, M.W., Igarashi, Y., Asakawa, S., Wong, L.L. (2019). Fine-scale population structure and ecotypes of anadromous Hilsa shad (*Tenualosa ilisha*) across complex aquatic ecosystems revealed by NextRAD genotyping. *Scientific Reports, 9,* 16050.

35. Xue, D.X., Yang, Q.L., Zong, S.B., Gao, T.X.., Liu, J.X. (2020). Genetic variation within and among range-wide populations of three ecotypes of the Japanese grenadier anchovy *Coilia nasus* with implications to its conservation and management. *Journal of Oceanology and Limnology, 38,* 851-861.

36. Duranton, M., Bonhomme, F., Gagnaire, P.-A. (2019). The spatial scale of dispersal revealed by admixture tracts. *Evolutionary Applications, 12,* 1743-1756.

37. Árnyas, E., Bereczki J, Tóth, A., Pecsenye, K., Varga, Z. (2006). Egg-laying preferences of the xerophilous ecotype of *Maculinea alcon* (Lepidoptera: Lycaenidae) in the Aggtelek National Park. *European Journal of Entomology, 103,* 587-595.

38. Espinosa, F., Ozawa, T. (2006). Population genetics of the endangered limpet *Patella ferruginea* (Gastropoda: Patellidae): taxonomic, conservation and evolutionary considerations. *Journal of Zoological Systematics and Evolutionary Research, 44,* 8-16.

39. Schönrogge, K., Barr, B., Wardlaw, J.C., Napper, E., Gardner, M.G., Breen, J., Elmes, G.W., Thomas, J.A. (2002). When rare species become endangered: cryptic speciation in myrmecophilous hoverflies. *Biological Journal of the Linnean Society, 75,* 291-300.

40. Osvath-Ferencz, M., Czekes, Z., Molnar, G., Marko, B., Vizauer, T.C., Rakosy, L., Nowicki, P. (2016). Adult population ecology and egg laying strategy in the 'cruciata' ecotype of the endangered butterfly *Maculinea alcon* (Lepidoptera: Lycaenidae). *Journal of Insect Conservation, 20,* 255-264.

41. Meriste, M., Helm, A., Ivask, M. (2016). Ground-dwelling spider fauna of flooded meadows in Matsalu, Estonia. *Wetlands, 36,* 525-537.

42. Keller, E.M., Harris, I., Cross, P. (2014). Identifying suitable queen rearing sites of *Apis mellifera mellifera* at a regional scale using morphometrics. *Journal of Apicultural Research, 53,* 279-287.

43. Green, B.S., Pederson, H., Gardner, C. (2013). Overlap of home ranges of resident and introduced southern rock lobster after translocation. *Reviews in Fisheries Science, 21*, 258-266.

44. Bonn, A., Schröder, B. (2001). Habitat models and their transfer for single and multi species groups: a case study of carabids in an alluvial forest. *Ecography, 24*, 483-496.

45. Hajek, M., Hajkova, P., Apostolova, I., Horsak, M., Rozbrojova, Z., Sopotlieva, D., Velev, N. (2010). The insecure future of Bulgarian refugial mires: economic progress versus Natura 2000. *Oryx, 44*, 539-546.

46. Baldivieso-Freitas, P., Blanco-Moreno, J.M., Gutiérrez-López, M., Peigné, J., Pérez-Ferrer, A., Trigo-Aza, D., Francesc Xavier Sansa, F.X. (2018). Earthworm abundance response to conservation agriculture practices in organic arable farming under Mediterranean climate. *Pedobiologia - Journal of Soil Ecology, 66*, 58-64.

47. Parsons, K.M., Durban, J.W., Burdin, A.M., Burkanov, V.N., Pitman, R.L., Barlow, J., … Wade, P.R. (2013). Geographic patterns of genetic differentiation among killer whales in the Northern North Pacific. *Journal of Heredity, 104*, 737-754.

48. Caballero, S., Islas-Villanueva, V., Tezanos-Pinto, G., Duchene, S., Delgado-Estrella, A., Sanchez-Okrucky, R., Mignucci-Giannoni, A.A. (2012). Phylogeography, genetic diversity and population structure of common bottlenose dolphins in the Wider Caribbean inferred from analyses of mitochondrial DNA control region sequences and microsatellite loci: Conservation and management implications. *Animal Conservation, 15*, 95-112.

49. Nagy, J.A., Johnson, D.L., Larter, N.C., Campbell, M.W., Derocher, A.E., Kelly, A., Dumond, M., Allaire, D., Croft, B. (2011). Subpopulation structure of caribou (*Rangifer tarandus* L.) in arctic and subarctic Canada. *Ecological Applications, 21*, 2334-2348.

50. McCafferty, D.J., Parsons, E.C.M. (2011). Marine mammal ecotypes: Implications for otter conservation and management. *Aquatic Mammals, 37*, 205-207.

51. Morin, P.A., Archer, F.I., Foote, A.D., Vilstrup, J., Allen, E.E., Wade, P., … Harkins, T. (2010). Complete mitochondrial genome phylogeographic analysis of killer whales (*Orcinus orca*) indicates multiple species. *Genome Research, 20*, 908-916.

52. Riccialdelli, L., Newsome, S.D., Fogel, M.L., Goodall, R.N.P. (2010). Isotopic assessment of prey and habitat preferences of a cetacean community in the southwestern South Atlantic Ocean. *Marine Ecology Progress Series, 418*, 235-248.

53. Wittmer, H.U., McLellan, B.N., Serrouya, R., Apps, C.D. (2007). Changes in landscape composition influence the decline of a threatened woodland caribou population. *Journal of Animal Ecology, 76*, 568-579.

54. Segura, I., Rocha-Olivares, A., Flores-Ramirez, S., Rojas-Bracho, L. (2006). Conservation implications of the genetic and ecological distinction of *Tursiops truncatus* ecotypes in the Gulf of California. *Biological Conservation, 133*, 336-346.

55. Parsons, K.M., Durban, J.W., Claridge, D.E., Herzing, D.L., Balcomb, K.C., Noble, L.R. (2006). Population genetic structure of coastal bottlenose dolphins (*Tursiops truncatus*) in the Northern Bahamas. *Marine Mammal Science, 22*, 276-298.

56. Ford, J.K.B., Ellis, G.M. (2006). Selective foraging by fish-eating killer whales *Orcinus orca* in British Columbia. *Marine Ecology Progress Series, 316*, 185-199.

57. Hinkes, M.T., Collins, G.H., Van Daele, L.J., Kovach, S.D., Aderman, A.R., Woolington, J.D., Seavoy, R.J. (2005). Influence of population growth on caribou herd identity, calving ground fidelity, and behavior. *Journal of Wildlife Management, 69,* 1147-1162.

58. Wittmer, H.U., McLellan, B.N., Seip, D.R., Young, J.A., Kinley, T.A., Watts, G.S., Hamilton, D. (2005). Population dynamics of the endangered mountain ecotype of woodland caribou (*Rangifer tarandus caribou*) in British Columbia, Canada. *Canadian Journal of Zoology, 83,* 407-418.

59. Courtois, R., Ouellet, J.P., Dussault, C., Gingras, A. (2004). Forest management guidelines for forest-dwelling caribou in Quebec. *Forestry Chronicle, 80,* 598-607.

60. Courtois, R., Bernatchez, L., Ouellet, J.P., Breton, L. (2003). Significance of caribou (*Rangifer tarandus*) ecotypes from a molecular genetics viewpoint. *Conservation Genetics, 4,* 393-404.

61. Bain, K., Wayne, A., Bencini, R. (2015). Risks in extrapolating habitat preferences over the geographical range of threatened taxa: a case study of the quokka (*Setonix brachyurus*) in the southern forests of Western Australia. *Wildlife Research, 42,* 334-342.

62. Wiedmann, B.P., Sargeant, G.A. (2014). Ecotypic variation in recruitment of reintroduced bighorn sheep: Implications for translocation. *Journal of Wildlife Management, 78,* 394-401.

63. Beerman, A., Ashe, E., Preedy, K., Williams, R. (2016). Sexual segregation when foraging in an extremely social killer whale population. *Behavioral Ecology and Sociobiology, 70,* 189-198.

64. Martinez, A.M., Gama, L.T., Delgado, J.V., Canon, J., Amills, M., de Sousa, C.B., … Sevane N. (2015). The Southwestern fringe of Europe as an important reservoir of caprine biodiversity. *Genetics Selection Evolution, 47,* 86.

65. Shafer, A.B.A., Nielsen, S.E., Northrup, J.M., Stenhouse, G.B. (2014). Linking genotype, ecotype, and phenotype in an intensively managed large carnivore. *Evolutionary Applications, 7,* 301-312.

66. Klütsch, C.F.C., Manseau, M., Trim, V., Polfus, J., Wilson, P.J. (2016). The eastern migratory caribou: the role of genetic introgression in ecotype evolution. *Royal Society Open Science, 3,* 150469.

67. Yannic, G., St-Laurent, M.-H., Ortego, J., Taillon, J., Beauchemin, A., Bernatchez, L., Dussault, C., Côté, S.D. (2016). Integrating ecological and genetic structure to define management units for caribou in Eastern Canada. *Conservation Genetics, 17,* 437-453.

68. Denryter, K.A., Cook, R.C., Cook, J.G., Parker, K.L. (2017). Straight from the caribou's (*Rangifer tarandus*) mouth: detailed observations of tame caribou reveal new insights into summer-autumn diets. *Canadian Journal of Zoology, 95,* 81-94.

69. Theberge, JB. (1991). Ecological classification, status and management of the gray wolf, *Canis lupus,* in Canada. *Canadian Field-Naturalist, 105,* 459-463.

70. Hendricks, S.A., Schweizer, R.M., Wayne, R.K. (2019). Conservation genomics illuminates the adaptive uniqueness of North American gray wolves. *Conservation Genetics, 20,* 29-43.

71. Priadka, P., Manseau, M., Trottier, T., Hervieux, D., Galpern, P., McLoughlin, P.D., Wilson, P.J. (2019). Partitioning drivers of spatial genetic variation for a continuously distributed population of boreal caribou: Implications for management unit delineation. *Ecology and Evolution, 9,* 141-153.

72. Tavares, S.B., Samarra, F.I.P., Pascoal, S., Graves, J.A., Miller, P.J.O. (2018). Killer whales (*Orcinus orca*) in Iceland show weak genetic structure among diverse isotopic signatures and observed movement patterns. *Ecology and Evolution, 8,* 11900-11913.

73. Bayas-Rea, R.L.Á., Félix, F., Montufar, R. (2018). Genetic divergence and fine scale population structure of the common bottlenose dolphin (*Tursiops truncatus*, Montagu) found in the Gulf of Guayaquil, Ecuador. *PeerJ, 6,* e4589.

74. Hulva, P., Bolfíková, B.Č., Woznicová, W., Jindřichová, M., Benešová, M., Mysłajek, R.W., … Antal, V. (2018). Wolves at the crossroads: fission-fusion range biography in the Western Carpathians and Central Europe. *Diversity and Distributions, 24,* 179-192.

75. Nykänen, M., Louis, M., Dillane, E., Alfonsi, E., Berrow, S., O'Brien, J. (2019). Fine-scale population structure and connectivity of bottlenose dolphins, *Tursiops truncatus*, in European waters and implications for conservation. *Aquatic Conservation: Marine and Freshwater Ecosystems, 29,* 197-211.

76. Fumagalli, M., Cesario, A., Costa, M. (2019). Where dolphins sleep: resting areas in the Red Sea. In: N.M.A. Rasul & I.C.F. Stewart (Eds.), Oceanographic and Biological aspects of the Red Sea (pp. 305-326). Springer Oceanography, Springer, Cham. doi: 10.1007/978-3-319-99417-8.

77. Fandohan, B., Gouwakinnou, G.N., Fonton, N.H., Sinsin, B., Liu, J. (2013). Impact of climate change on the geographical distribution of suitable areas for cultivation and conservation of underutilized fruit trees: case study of the tamarind tree in Benin. *Biotechnologie, Agronomie, Société et Environnement, 17,* 450-462.

78. Ratnadass, A., Wink, M. (2012). The phorbol ester fraction from *Jatropha curcas* seed oil: Potential and limits for crop protection against insect pests. *International Journal of Molecular Sciences, 13,* 16157-16171.

79. Mutegi, E., Sagnard, F., Muraya, M., Kanyenji, B., Rono, B., Mwongera, C., … Labuschagne, M. (2010). Ecogeographical distribution of wild, weedy and cultivated *Sorghum bicolor* (L.) Moench in Kenya: implications for conservation and crop-to-wild gene flow. *Genetic Resources and Crop Evolution, 57,* 243-253.

80. Boller, B., Veteläinen, M. (2010). A state of the art of germplasm collections for forage and turf species. In: C. Huyghe (Ed.), Sustainable use of genetic diversity in forage and turf breeding (pp. 17-28). Dordrecht: Springer, doi: 10.1007/978-90-481-8706-5.

81. Castillo, C., Sotomayor, L., Ortiz, C., Leonelli, G., Borie, F., Rubio, R. (2009). Effects of Arbuscular Mycorrhizal fungi on an ecological crop of chili peppers (*Capsicum annuum* L.). *Chilean Journal of Agricultural Research, 69,* 79-87.

82. Kolliker, R., Boller, B., Majidi, M., Peter-Schmid, M.K.I., Bassin, S., Widmer, F. (2009). Characterization and utilization of genetic resources for improvement and management of grassland species. In: Y. Yamada & G. Spangenberg (Eds.), Molecular Breeding of Forage and Turf (pp. 55-70). New York: Springer, doi: 10.1007/978-0-387-79144-9_5.

83. Leibing, C., Zonneveld, M., Jarvis, A., Dvorak, W. (2009). Adaptation of tropical and subtropical pine plantation forestry to climate change: Realignment of *Pinus patula* and *Pinus tecunumanii* genotypes to 2020 planting site climates. *Scandinavian Journal of Forest Research, 24,* 483-493.

84. Bacchetta, L., Avanzato, D., Botta, R., Boccacci, P., Drogoudi, P., Metzidakis, I., … Di Giovanni B. (2009). First results of "Safenut": a European project for the preservation and utilization of hazelnut local genetic resources. VII International Congress on Hazelnut. 1 October 2009, Viterbo, Italy.

85. Peter-Schmid, M.K.I., Kolliker, R., Boller, B. (2008). Value of permanent grassland habitats as reservoirs of *Festuca pratensis* Huds. and *Lolium multiflorum* Lam. populations for breeding and conservation. *Euphytica, 164,* 239-253.

86. Peter-Schmid, M.K.I., Boller, B., Kolliker, R. (2008). Habitat and management affect genetic structure of *Festuca pratensis* but not *Lolium multiflorum* ecotype populations. *Plant Breeding, 127,* 510-517.

87. Ritterbusch, D. (2007). Growth patterns of reed (*Phragmites australis*): the development of reed stands in carp ponds. *Aquaculture International, 15,* 191-199.

88. Fischer, A.P., Bliss, J. (2006). Mental and biophysical terrains of biodiversity: Conserving oak on family forests. *Society and Natural Resources, 19,* 625-643.

89. St John, L., Blaker, P. (2004). New native plant releases from the USDA-NRCS, Aberdeen, ID, Plant Materials Center. In: A.L. Hild, L. Nancz, S.E. Mezer, E.W. Schupp, T. Booth, comps. Seed and Soil Dynamics in Shrubland Ecosystems: Proceedings 31: 138-139. Seed and Soil Dynamics in Shrubland Ecosystems Conference, 12-16 August 2002, Laramie, WY, USA.

90. Orgeas, J., Ourcival, J.M., Bonin, G. (2003). Seasonal and spatial patterns of foliar nutrients in cork oak (*Quercus suber* L.) growing on siliceous soils in Provence (France). *Plant Ecology, 164,* 201-211.

91. Menz, M.H.M., Phillips, R.D., Anthony, J.M., Bohman, B., Dixon, K.W., Peakall, R. (2015). Ecological and genetic evidence for cryptic ecotypes in a rare sexually deceptive orchid, *Drakaea elastic*. *Botanical Journal of the Linnean Society, 177*, 124-140.

92. Alam, M.K., Salahin, N., Islam, S., Begum, R.A., Hasanuzzaman, M., Islam, M.S., Rahman, M.M. (2017). Patterns of change in soil organic matter, physical properties and crop productivity under tillage practices and cropping systems in Bangladesh. *Journal of Agricultural Science, 155*, 216-238.

93. Blažek, P., Lepš, J., Fajmon, K., Těšitel, J. (2016). Response of two hemiparasitic Orobanchaceae species to mowing dates: implications for grassland conservation and restoration practice. Plant Ecology and Evolution, 149, 31-38.

94. Wiehle, M., Prinz, K., Kehlenbeck, K., Goenster, S., Mohamed, S.A., Finkeldey, R., Buerkert, A., Gebauer, J. (2014). The African baobab (*Adansonia digitata*, Malvaceae): Genetic resources in neglected populations of the Nuba Mountains, Sudan. *American Journal of Botany, 101*, 1498-1507.

95. Caturegli, L., Lulli, F., Foschi, L., Guglielminetti, L., Bonari, E., Volterrani, M. (2015). Turfgrass spectral reflectance: simulating satellite monitoring of spectral signatures of main C3 and C4 species. *Precision Agriculture, 16*, 297-310.

96. Abdou, R., Malice, M., Bakasso, Y., Saadou, M., Baudoin, J.P. (2014). Folk taxonomy of Niger onion diversity and analysis of farmer criteria for characterizing the different ecotypes. *Cahiers Agriculture*, *23*, 166-176.

97. Erickson, B., Navarrete-Tindall, N.E. (2004). Missouri Native Ecotype Program: Increasing local-source native seed. *Natural Areas Journal, 24,* 15-22.

98. Ferreira, M.T., Albuquerque, A., Aguiar, F.C., Sidorkewicz, N. (2002). Assessing reference sites and ecological quality of river plant assemblages from an Iberian basin using a multivariate approach. *Archiv für Hydrobiologie, 155,* 121-145.

99. St John, L., Blaker, P. (2001). New plant releases from the USDA-NRCS Aberdeen, Idaho, Plant Materials Center. In: McArthur, E. Durant; Fairbanks, Daniel J., comps. Shrubland Ecosystem Genetics and Biodiversity: Proceedings 21: 143-144. June 13–15, Provo, UT. Proc. RMRS-P-21. Ogden, UT: U.S. Department of Agriculture, Forest Service, Rocky Mountain Research Station.

100. West, N.E. (1993). Biodiversity of rangelands. Journal of Range Management 46: 2-13.

101. Khadivi, A., Gismondi, A., Canini, A. (2019). Genetic characterization of Iranian grapes (*Vitis vinifera* L.) and their relationships with Italian ecotypes. *Agroforestry Systems, 93*, 435-447.

102. Macdonald, S., Bailey, T., Hunt, M., Davidson, N., Jordan, G. (2019). Stable states in soil chemistry persist in eucalypt woodland restorations. *Applied Vegetation Science, 22*, 105-114.

103. Yuan, X.Y., Sun, Y.W., Bai, X.R., Dang, M., Feng, X.J., Zulfiqar, S., Zhao, P. (2018). Population structure, genetic diversity, and gene introgression of two closely related walnuts (*Juglans regia* and *J. sigillata*) in Southwestern China revealed by EST-SSR markers. Forests, 9(10), 646.

104. Bozchaloyi, S.E., Sheidai, M. (2018). Molecular diversity and genetic relationships among Geranium pusillum and G. pyrenaicum with inter simple sequence repeat (ISSR) regions. *Caryologia, 71*, 457-470.

105. Altrichter, E.A., Thompson, J.R., Mabry, C.M. (2017). Stakeholders' perceptions of native plants and local ecotypes in ecological restoration. *Ecological Restoration, 35*, 218-228.

106. Dobrowolska, D., Bončina, A., Klumpp, R. (2017). Ecology and silviculture of silver fir (*Abies alba* Mill.): a review. *Journal of Forest Research, 22*, 326-335.

107. Berend, K., Haynes, K., MacKenzie, C. M. (2019). Common garden experiments as a dynamic tool for ecological studies of alpine plants and communities in northeastern North America. *Rhodora, 121*, 174-212.

108. Gaos, A.R., Lewison, R.L., Liles, M.J., Gadea, V., Altamirano, E., Henriquez, A.V., … Dutton, P.H. (2016). Hawksbill turtle terra incognita: conservation genetics of eastern Pacific rookeries. *Ecology and Evolution, 6*, 1251-1264.

109. Freitas, I., Fahd, S., Velo-Antón G., Martínez-Freiría, F. (2018). Chasing the phantom: biogeography and conservation of *Vipera latastei-monticola* in the Maghreb (North Africa). *Amphibia-Reptilia, 39*, 145-161.

110. Fourcade, Y., Chaput-Bardy, A., Secondi, J., Fleurant, C., Lemaire, C. (2013). Is local selection so widespread in river organisms? Fractal geometry of river networks leads to high bias in outlier detection. *Molecular Ecology, 22*, 2065-2073.

111. Morrison, M.L. (2012). The habitat sampling and analysis paradigm has limited value in animal conservation: A prequel. *Journal of Wildlife Management, 76*, 438-450.

112. Kreyling, J., Bittner, T., Jaeschke, A., Jentsch, A., Steinbauer, M.J., Thiel, D., Beierkuhnlein, C. (2011). Assisted colonization: A question of focal units and recipient localities. *Restoration Ecology, 19*, 433-440.

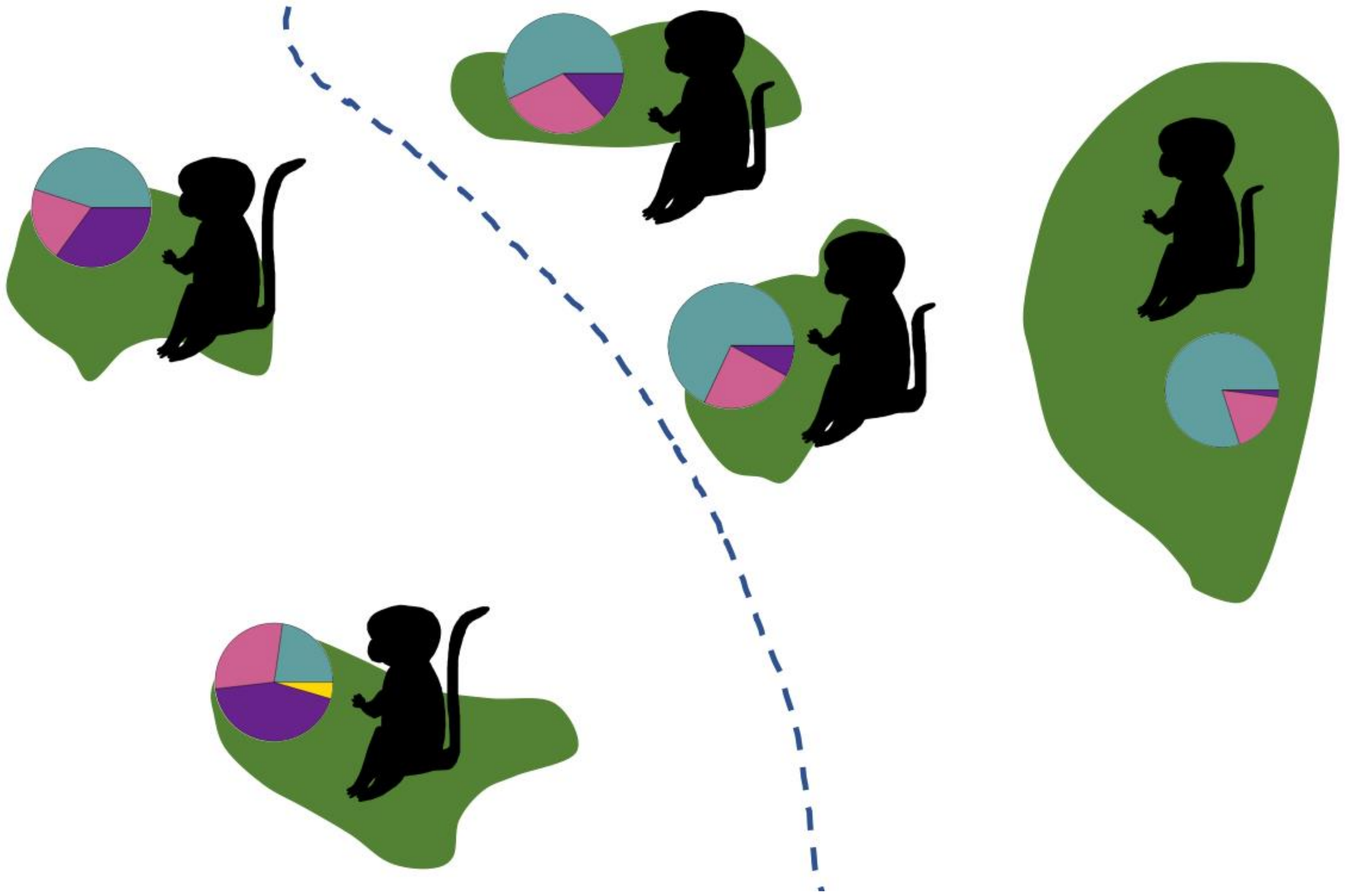

Figure S1. A hypothetical meta-population of a monkey split into two morphs: long-tailed and short-tailed. The dashed line suggests two distinct populations based on ecotype. The pie charts show the genetic structure of each subpopulation. The ecotype distinction and the genetic structure suggest two different histories, with the southern-most population containing unique ancestry (yellow portion of the pie).

Supplemental Note S1. Taxonomy

Careful delineation of taxonomic groups is important because these classifications have long-term implications for conservation efforts (Luo et al., 2018), and such intraspecific units represent important evolutionary potential within a species. Yet confounding – or a lack of – clear criteria to categorise these entities can seriously delay or limit conservation action, also because management agencies sometimes lack the taxonomic expertise to assess conflicting scientific assessments (Haig et al., 2006). Scientists should likewise be mindful of the importance of translating new evidence for genetically defined units into categories that can be considered under local legislation, while communicating the nuances of speciation that is emerging from genomic research (Coates et al., 2018). Defining excessively broad taxonomic units can lead to management actions resulting in outbreeding depression, whereas taxonomic units that are too narrow risk management actions that lead to inbreeding depression (Frankham et al., 2012). Frankham et al. (2012) and Haig et al. (2006) have therefore suggested that taxonomic splitting with the aim of conserving more biodiversity can actually hinder conservation aimed at preserving small populations, resulting in a loss in biodiversity over time (but see Gippoliti & Groves, 2012 for a different view). There are long-held concerns that scientific evidence behind taxonomic distinction should be clear and transparent, to avoid concerns over 'taxonomic inflation', which can affect conservation priorities and actions (Berta & Churchill, 2012). Oftentimes, there are clear links between systematic rankings and priorities for preservation (Stanton et al., 2019), whereby reassignment to a lower status can be deemed unfavourable for conservation (Bowen & Karl 1999; Crandall et al., 2000). Several authors have highlighted that, although taxonomic units should influence conservation strategies, conservation strategies ought not to influence taxonomic decisions (Bowen & Karl, 1999; Haig et al., 2006). Moreover, taxonomic status assessments, like that of other adaptive management, may need periodic re-evaluation to consider new knowledge and technical developments (Ford, 2004; Haig et al., 2006) and the same

may be relevant for conservation of intraspecific variability. Multiple lines of evidence should be considered for taxonomic decisions (Stanton et al., 2019), and this approach is also likely to benefit assessments of ecotypes.

Supplemental Note S2. Caribou (*Rangifer tarandus*) ecotypes

The influence of sporadic gene flow among reported caribou ecotypes remains unresolved (Courtois et al., 2003) and may be affected by various factors including evolutionary history, effective population size, and habitat connectivity (Courtois et al., 2003; Klütsch et al., 2016; Serrouya et al., 2012; Yannic et al., 2017). The terminology used to describe caribou ecotypes is often unclear (COSEWIC, 2011), and from a practical management perspective, low confidence in individual caribou assignment to specific ecotypes have limited its application in law enforcement (Bourret et al., 2020). Microsatellite studies have recently reported conservation units identified by genetic and geographical structure (Jenkins et al. 2018), and a wide-scale investigation across eastern Canada by Klütsch et al. (2016) found overall broad agreement between genetic structure and boreal vs. eastern migratory ecotypes. Moreover, new results from genome-wide analyses indicate parallel evolution of similar ecotypes from separate evolutionary lineages and suggest that the designation of caribou conservation units merits further revision (Taylor et al., 2020). Findings for this highly mobile species with circumpolar distribution have so far been focused mainly on neutral genetic markers (Yannic et al., 2017) which have been the available resources for genetic analyses, but see Cavedon et al. (2019), Kharzinova et al. (2016), Shafer et al. (2016), Taylor et al. (2019, 2020), and Weldenegodguad et al. (2020) for new genomic findings and broader discussions around their interpretation. Even so, studies at various spatial scales have revealed discrepancies between differentiation based on (putatively) neutral genetic markers and ecological units (see illustration in Supplemental Figure S1) and highlight the need for further research on adaptive genetic

differentiation (Taylor et al., 2020; Yannic et al., 2016, 2017). Hence, the case of *Rangifer tarandus* (including North American caribou and Eurasian reindeer) illustrates how new genomic results can help illuminate the spatiotemporal distribution of adaptive (functional) genetic variation within a species (Box 1 Part B) and, over time, inform conservation units that integrate emerging knowledge of ecological and evolutionary genetics. Future study of functional genetic variation could provide key information on natural selection and adaptation, which is increasingly important for long-term persistence in environments rapidly altered by climate change (Sgrò et al., 2011; Yannic et al., 2017), and data on such variation may also help delineate caribou designatable units and ecotypes (COSEWIC, 2011).

Supplemental Note S3. Analytical considerations

Spatial structure and assumptions of genetic equilibrium conditions. Improved understanding of contemporary gene flow and genetic structure can provide important real-time information for conservation management of different ecotypes (Dahle et al., 2018). Contemporary gene flow may be investigated looking at carefully designed marker panels (high minor allele frequency (MAF), unlinked loci) and sampling (homogeneous and high density) optimized for assessing relatedness across the landscape that does not require assumptions based on equilibrium populations (Norman et al., 2017). Here, the degree of "landscape relatedness" will provide a spatial measure of the extent of relatedness among individuals on the landscape. However, whereas standard measures in population genetics will only integrate those movements that cause meaningful population effects (Cushman et al., 2006), landscape relatedness estimates do not assume that animals with low levels of relatedness to individuals sampled nearby have dispersed. Selection can occur in the presence of gene flow (Fitzpatrick et al., 2015), which has the potential to confound our interpretations. Various

complementary methods can therefore be used to highlight different spatiotemporal processes that matter for monitoring genetic diversity and landscape connectivity.

A key issue for future genetic analyses will be to harness the information from analyses with different markers, sampling areas, time spans, and objectives to extract as much information as possible on the influence of various spatiotemporal processes (de Groot et al., 2016). At the individual level, dispersal decisions could be influenced by the extent to which surrounding areas are occupied by relatives (Zedrosser et al., 2007) and whether habitat resembles that of the native range (Edelaar et al., 2008; Sacks et al., 2004). Certain species inhabit a temporary landscape with moving territories, represented by polar bears (*Ursus maritimus*) (Kutschera et al., 2016) as well as Arctic foxes (*Vulpes lagopus*) and gray wolves (*Canis lupus*) in some areas (Carmichael et al., 2007). In wolves, dietary specialization may explain seasonal migrations over large areas in certain regions (Musiani et al., 2007) and genetic differentiation across short distances in others (Pilot et al., 2012; Stronen et al., 2014). Here landscape ecology and inherent social behaviour interact to produce location-specific hierarchies of spatial genetic structure. A study of a given species in one landscape may therefore find that a certain variable has a large effect on genetic differentiation, whereas its effects may appear much weaker and relatively less important elsewhere, which can be explained by differences in the degree to which the landscape composition and configuration limit gene flow (Cushman et al., 2013). In addition, history and biogeography can at times provide a better explanation for observed patterns than ecological or evolutionary developments (Warren et al., 2014). Sampling may also follow political boundaries that have no biological relevance; perhaps producing inflated population estimates (Bischof et al., 2016) or more genetic clusters than what an organized sampling through the overall range of the population would show. For wide-ranging species, the availability of “hard data” that e.g., confirms collared (or similarly tagged) animals traveling across heavily human-altered

landscapes (Ciucci et al., 2009) and remarkable long-distance movements that can involve important ecological changes (Fuglei & Tarroux, 2019) also help limit speculation about wide-ranging wildlife species and promote science-based management involving different jurisdictions (Ciucci et al., 2009).

Furthermore, spatial genetic structure may coincide with natural landscape transitions without necessarily being shaped or influenced by these features (Bierne et al., 2011). Similarly, landscape and/or ecological transitions that occur naturally (i.e., independent from human activities) can result in spatial genetic structure (Muñoz-Fuentes et al., 2009; Pilot et al., 2012). The presence of such structure could easily remain unnoticed; although at times it may be well known to those intimately tied to the local natural history, for instance, local indigenous communities (Stronen et al., 2014). Current genetic structures can also reflect historical landscape features that no longer exist, exemplified by the corridor that permitted brown bears (*U. arctos*) from the Iberian lineage entry to the Scandinavia peninsula following retreat of the ice sheet after the last glaciation (Bray et al., 2013). In contrast, the genetic structure of a fast-evolving virus has been used to infer population genetic structure of its large carnivore host over a more recent time scale (Biek et al., 2006).

More attention toward environmental variation and selection. New methods have enabled the addition of new evolutionary perspectives to the classic approaches primarily focused on neutral genetic markers. However, even studies that genotype thousands of individuals at a large number of SNPs generally uncover only a small proportion of the heritable variation of a trait (Meirmans, 2015). The next step is improved design of studies that will enhance our ability to detect this genetic variation, especially in the presence of seemingly contradictory animal movement (Moura et al., 2014; Riley et al., 2006; Vander Wal et al., 2013). A further aim is to recognize the selective pressures driving genetic changes and to identify how and where genetic differences are expressed within the

genome. With large numbers of markers, completely avoiding false positives may be almost impossible. Adjustment of alpha levels or correction for multiple testing may help to a degree, but at the cost of inflating type II errors (Meirmans, 2015). Multiple testing approaches might help, but could lead to biases, at least in part, by the particularities and strengths of the given method (Narum & Hess, 2011; Stucki et al., 2017). A combination of methods may highlight genes, environmental factors, and/or potential driving processes behind genetic variation, which can point to priority areas for further investigation. Because various factors can confound the identification of ecotypes and their spatial distribution, it is important to consider that false-positive associations can occur if models fail to incorporate factors such as isolation by distance, shared demographic history, and cryptic relatedness (François et al., 2016). With an expanding toolbox of powerful techniques for genomic data analyses, careful attention is required to avoid overfitting models, while ensuring that they realistically capture the underlying biology (Liberles et al., 2013). A very small bias may have negligible effect in a microsatellite data set but become a strongly significant pattern in a next-generation sequencing project, which can cause overconfidence in the patterns observed (Meirmans, 2015). Conversely, a bias against negative results could mean that investigations not producing clear genetic structures are less likely to be published or undertaken, despite their importance for the public record. Theoretical investigations predicting and then simulating at what sample size or number of markers family structure and other effects will no longer be detected (or strongly influential) could provide a measure of sensitivity analysis to guide sampling design.

When interpreting genetic results, focusing more on biological relevance than on statistical significance is important; in the genomics era with thousands of loci, strong significance is easily obtained even for biologically marginal processes (Meirmans, 2015). Broad-scale landscape genetic studies must be carefully interpreted to avoid unwarranted extrapolation but provide key opportunities

for illuminating patterns and processes across geographical space and taxonomic groups (Richardson et al., 2016). From an ecological perspective, however, shifting the focus from the exact genes involved in adaptation toward the processes and environmental variables that lead to adaptation to the local climate could be valuable (Meirmans, 2015). This could aid investigation on the presence of local ecotypes, where multiple genetic variants may be under influence of the environmental and ecological forces under study. This approach may also help solve the problems related to having low statistical power. Especially in non-model organisms, the lack of statistical power can make it difficult to establish clear associations between adaptive genetic variation, phenotypes, and reproductive isolation (Foote, 2012). Past signatures of selection are not certain to indicate future adaptive potential, and it can often be difficult to determine the specific cause of selection (Stanton et al., 2019). Even if we have incomplete understanding of the underlying processes, identification of seemingly important genes can provide key advances for follow-up research and could further knowledge of connections among genes, communities, and ecological processes (Coulson et al., 2006). Good hypotheses often exist about climatic variables that may affect the study species, and there are usually fewer climatic variables than genes, leading to less testing and less need for correction (Meirmans, 2015). The emerging findings from genome-wide studies combined with sound ecological data provide rich opportunities to inform our understanding of biology and evolution, and can help us in setting thoughtful priorities for conservation at the species level and beyond. Moreover, data on responses to human activity following colonization of new habitats (Gompper, 2002; McPhearson et al., 2016), or responses to the local absence of predators (or competitors) and their possible implications for source-sink dynamics (Goldberg et al., 2014) could inform interpretations of genetic results and research design. Ecotypes may emerge and disappear following landscape and environmental alterations. Traits such as body size, which can show clear genetic and environmental components (Rivrud et al., 2019) and appear instrumental in ecological

genetic differentiation (Kays et al., 2010; Monzón et al., 2014) are often polygenic traits involving many genes each with a small effect. Accordingly, target genes may be difficult to detect with standard genome-wide association studies, although such traits could play a more important role in selection than that documented at present (Exposito-Alonso et al., 2018; François et al., 2016).

References

Berta, A., Churchill, M. (2012). Pinniped taxonomy: review of currently recognized species and subspecies, and evidence used for their description. *Mammal Review, 42*, 207-234.

Biek, R., Drummond, A.J., Poss, M. (2006). A virus reveals population structure and recent demographic history of its carnivore host. *Science, 311*, 538-541.

Bierne, N., Welch, J., Loire, E., Bonhomme, F., David, P. (2011). The coupling hypothesis: why genome scans may fail to map local adaptation genes. *Molecular Ecology, 20*, 2044-2072.

Bischof, R., Brøseth, H., Gimenez, O. (2016). Wildlife in a politically divided world: insularism inflates estimates of brown bear abundance. *Conservation Letters, 9*, 122-130.

Bourret, V., Albert, V., April, J., Côté, G., Morissette, O. (2020). Past, present and future contributions of evolutionary biology to wildlife forensics, management and conservation. *Evolutionary Applications, 13*, 1420-1434.

Bowen, B.W., Karl, S.A. (1999). In war, truth is the first casualty. *Conservation Biology, 13*, 1013-1016.

Bray, S.C.E., Austrin, J.J., Metcalf, J.L., Østbye, K., Østbye, E., Lauritzen, S.-E., … Cooper, A. (2013). Ancient DNA identifies post-glacial recolonisation, not recent bottlenecks, as the primary driver of contemporary mtDNA phylogeography and diversity in Scandinavian brown bears. *Diversity and Distributions, 19*, 245-256.

Carmichael, L.E., Krizan, J., Nagy, J.A., Fuglei, E., Dumond, M., Johnson, D., Veitch, A., Berteaux, D., Strobeck, C. (2007). Historical and ecological determinants of genetic structure in arctic canids. *Molecular Ecology, 16*, 3466-3483.

Cavedon, M., Gubili, C., Heppenheimer, E., vonHoldt, B., Mariani, S., Hebblewhite, M., … Musiani M. (2019). Genomics, environment and balancing selection in behaviourally bimodal populations: The caribou case. *Molecular Ecology, 28*, 1946-1963.

Ciucci, P., Reggioni, W., Maiorano, L., Boitani, L. (2009). Long-distance dispersal of a rescued wolf from the Northern Apennines to the Western Alps. *Journal of Wildlife Management, 73,* 1300-1306.

Coates, D.J., Byrne, M., Moritz, C. (2018). Genetic diversity and conservation units: Dealing with the species-population continuum in the age of genomics. *Frontiers in Ecology and Evolution, 6*, 165.

COSEWIC. (2011). Designatable Units for Caribou (*Rangifer tarandus*) in Canada. Committee on the Status of Endangered Wildlife in Canada. Ottawa. 88 pp.

Coulson, T., Benton, T.G., Lundberg, P., Dall, S.R.X., Kendall, B.E. (2006). Putting evolutionary biology back in the ecological theatre: a demographic framework mapping genes to communities. *Evolutionary Ecology Research, 8,* 1155-1171.

Courtois, R., Bernatchez, L., Ouellet, J.-P., Breton, L. (2003). Significance of caribou (*Rangifer tarandus*) ecotypes from a molecular genetics viewpoint. *Conservation Genetics, 4*, 393-404.

Crandall, K.A., Bininda-Emonds, O.R., Mace, G.M., Wayne, R.K. (2000). Considering evolutionary processes in conservation biology. *Trends in Ecology & Evolution, 15*, 290-295.

Cushman, S.A., McKelvey, K.S., Hayden, J., Schwartz, M.K. (2006). Gene-flow in complex landscapes: testing multiple models with causal modeling. *American Naturalist, 168,* 486-499.

Cushman, S.A., Shirt, A.J., Landguth, E.L. (2013). Landscape genetics and limiting factors. *Conservation Genetics, 14,* 263-274.

Dahle, G., Johansen, T., Westgaard, J.-I., Aglen, A., Glover, K.A. (2018). Genetic management of mixed-stock fisheries “real-time”: The case of the largest remaining cod fishery operating in the Atlantic in 2007–2017. *Fisheries Research, 205,* 77-85.

de Groot, G. A., Nowak, C., Skrbinšek, T., Andersen, L. W., Aspi, J., Fumagalli, L., … Muñoz-Fuentes, V. (2016). Decades of population genetic research reveal the need for harmonization of molecular markers: the grey wolf *Canis lupus* as a case study. *Mammal Review, 46*, 44-59.

Edelaar, P., Siepielski, A.M., Clobert, J. (2008). Matching habitat choice causes direct gene flow: a neglected dimension in evolution and ecology. *Evolution, 62*, 2462-2472.

Exposito-Alonso, M., Vasseur, F., Ding, W., Wang, G., Burbano, H.A., Weigel, D. (2018). Genomic basis and evolutionary potential for extreme drought adaptation in Arabidopsis thaliana. *Nature Ecology & Evolution, 2*, 352-358.

Fitzpatrick, S.W., Gerberich, J.C., Kronenberger, J.A., Angeloni, L.M., Funk, W.C. (2015). Locally adapted traits maintained in the face of high gene flow. *Ecology Letters, 18*, 37-47.

François, O., Martins, H., Caye, K., Schoville, S.D. (2016). Controlling false discoveries in genome scans for selection. *Molecular Ecology, 25*, 454-469.

Frankham, R., Ballou, J.D., Dudash, M.R., Eldridge, M.D.B., Fenster, C.B., Lacy, R.C., … Ryder, O.A. (2012). Implications of different species concepts for conserving biodiversity. *Biological Conservation, 153*, 25-31.

Foote, A.D. (2012). Investigating ecological speciation in non-model organisms: a case study of killer whale ecotypes. *Evolutionary Ecology Research, 14*, 447-465.

Ford, M.J. (2004). Conservation units and preserving diversity. In: A.P. Hendry & S.C. Stearns (Eds.), Evolution Illuminated: Salmon and Their Relatives (pp. 338-357). New York: Oxford University Press.

Fuglei, E., Tarroux, A. (2019). Arctic fox dispersal from Svalbard to Canada: one female's long run across sea ice. *Polar Research, 38,* 3512.

Gippoliti, S., Groves, C.P. (2012). "Taxonomic inflation" in the historical context of mammalogy and conservation. *Hystrix, 23,* 8-11.

Goldberg, J.F., Hebblewhite, M., Bardsley, J. (2014). Consequences of a refuge for the predator-prey dynamics of a wolf-elk system in Banff National Park, Alberta, Canada. *PLoS ONE, 9(3),* e91417.

Gompper, M.E. (2002). Top carnivores in the suburbs? Ecological and conservation issues raised by colonization of Northeastern North America by coyotes. *BioScience, 52,* 185-190.

Haig, S.M., Beever, E.A., Chambers, S.M., Draheim, H.M., Dugger, B.D., Dunham, S., … Sheffield, L.M. (2006). Taxonomic considerations in listing subspecies under the U.S. Endangered Species Act. *Conservation Biology, 20,* 1584-1594.

Jenkins, D.A., Yannic, G., Schaefer, J.A., Conolly, J., Lecomte, N. (2018). Population structure of caribou in an ice-bound archipelago. *Diversity and Distributions, 24,* 1092-1108.

Kays, R., Curtis, A., Kirchman, J.J. (2010). Rapid adaptive evolution of northeastern coyotes via hybridization with wolves. *Biology Letters*, *23,* 89-93.

Kharzinova, V.R., Sermyagin, A.A., Gladyr, E.A., Okhlopkov, I.M., Brem, G., Zinovieva, N.A. (2015). A study of applicability of SNP chips developed for bovine and ovine species to whole-genome analysis of reindeer *Rangifer tarandus*. *Journal of Heredity*, *106,* 758-761.

Klütsch, C.F.C., Manseau, M., Trim, V., Polfus, J., Wilson, P.W. (2016). The eastern migratory caribou: the role of genetic introgression in ecotype evolution. *Royal Society Open Science, 3,* 150469.

Kutschera, V.E., Frosch, C., Janke, A., Skírnisson, K, Bidon, T., Lecomte, N., … Hailer, F. (2016). High genetic variability of vagrant polar bears illustrates importance of population connectivity in fragmented sea ice habitats. *Animal Conservation, 19,* 337-349.

Liberles, D.A., Teufel, A.I., Liu, L., Stadler, T. (2013). On the need for mechanistic models in computational genomics and metagenomics. *Genome Biology and Evolution, 5,* 2008-2018.

Luo, A., Ling, C., Ho, S.Y.W., Zhu, C.-D. (2018). Comparison of methods for molecular species delimitation across a range of speciation scenarios. *Systematic Biology, 67*, 830-846.

McPhearson, T., Pickett, S.T.A., Grimm, N.B., Niemelä, J., Alberti M, Elmquist T, … Qureshi S. (2016). Advancing urban ecology toward a science of cities. *BioScience, 66*, 198-212.

Meirmans, PG. (2015). Seven common mistakes in population genetics and how to avoid them. *Molecular Ecology, 24*, 3223-3231.

Monzón, J., Kays, R., Dykhuizen, D.E. (2014). Assessment of coyote-wolf-dog admixture using ancestry-informative diagnostic SNPs. *Molecular Ecology, 23*, 182-197.

Moura, A.E., Kenny, J.G., Chaudhuri, R., Hughes, M.A., Welch, A.J., Reisinger, R.R., … Hoelzel, R. (2014). Population genomics of the killer whale indicates ecotype evolution in sympatry involving both selection and drift. *Molecular Ecology, 23*, 5179-5192.

Muñoz-Fuentes, V., Darimont, C.T., Wayne, R.K., Paquet, P.C., Leonard, J.A. (2009). Ecological factors drive differentiation in wolves from British Columbia. *Journal of Biogeography, 36*, 1516-1531.

Musiani, M., Leonard, J.A., Cluff, H.D., Gates, C.C., Mariani, S., Paquet, P.C., Vilà, C., Wayne, R.K. (2007). Differentiation of tundra and boreal coniferous forest wolves: genetics, coat color and association with migratory caribou. *Molecular Ecology, 16*, 4149-4170.

Narum, S.R., Hess, J.E. (2011). Comparison of F_{ST} outlier tests for SNP loci under selection. *Molecular Ecology Resources, 11*, 184-194.

Norman, A.J., Stronen, A.V., Fuglstad, G.-A., Ruiz-Gonzales, A., Kindberg, J., Street, N.R., Spong, G. (2017). Landscape relatedness: Detecting contemporary, fine-scale spatial structure in wild populations. *Landscape Ecology, 32*, 181-194.

Pilot, M., Jędrzejewski, W., Sidorovich, V.E., Meier-Augustein, W., Hoelzel, A.R. (2012). Dietary differentiation and the evolution of population genetic structure in a highly mobile carnivore. *PLoS ONE, 7(6),* e39341.

Richardson, J.L., Brady, S.P., Wang, I.J., Spear, S.F. (2016). Navigating the pitfalls and promise of landscape genetics. *Molecular Ecology, 25,* 849-863.

Riley, S.P., Pollinger, J.P., Sauvajot, R.M., York, E.C., Bromley, C., Fuller, T.K., Wayne, R.K. (2006). A southern California freeway is a physical and social barrier to gene flow in carnivores. *Molecular Ecology, 15,* 1733-1741.

Rivrud, I.M., Frank, S.C., Bischof, R., Mysterud, A., Steyaert, S.M.J.G., Hertel, A.G., … Zedrosser, A. (2019). Heritability of head size in a hunted large carnivore, the brown bear (*Ursus arctos*). *Evolutionary Applications, 12,* 1124-1135.

Sacks, B.N., Brown, S.K., Ernest, H.B. (2004). Population structure of California coyotes corresponds to habitat-specific breaks and illuminates species history. *Molecular Ecology, 13,* 1265-1275.

Serrouya, R., Paetkau, D., McLellan, B.N., Boutin, S., Campbell, M., Jenkins, D.A. (2012). Population size and major valleys explain microsatellite variation better than taxonomic units for caribou in western Canada. *Molecular Ecology, 21,* 2588-2601.

Sgrò, C.M., Lowe, A.J., Hoffmann, A.A. (2011). Building evolutionary resilience for conserving biodiversity under climate change. *Evolutionary Applications, 4,* 326-337.

Shafer, A.B.A., Miller, J.M., Kardos, M. (2016). Cross-species application of SNP chips is not suitable for identifying runs of homozygosity. *Journal of Heredity, 107,* 193-195.

Stanton, D.W.G., Frandsen, P., Waples, R.K., Heller, R., Russo, I.-R.M., Orozco-terWengel, P.A., … Bruford, M.W. (2019). More grist for the mill? Species delimitation in the genomic era and its implications for conservation. *Conservation Genetics, 20,* 101-113.

Stronen, A.V., Navid, E.L., Quinn, M.S., Paquet, P.C., Bryan, H.M., Darimont, C.T. (2014). Population genetic structure of gray wolves (*Canis lupus*) in a marine archipelago suggests island-mainland differentiation consistent with dietary niche. *BMC Ecology, 14,* 11.

Stucki, S. Orozco-terWengel, P., Forester, B.R., Duruz, S., Colli, L., Masembe, C., … Joost, S. (2017). High performance computation of landscape genomic models including local indicators of spatial association. *Molecular Ecology Resources, 17,* 1072-1089.

Taylor, R.S., Horn, R.L., Zhang, X., Golding, G.B., Manseau, M., Wilson, P.J. (2019). The caribou (*Rangifer tarandus*) genome. *Genes, 10,* 540.

Taylor, R.S., Manseau, M., Horn, R.L., Keobouasone, S., Golding, G.B., Wilson, P.J. (2020). The role of introgression and ecotypic parallelism in delineating intraspecific conservation units. *Molecular Ecology, 29,* 2793-2809.

Vander Wal, E., Edye, I., Paquet, P.C., Coltman, D.W., Bayne, E., Brook, R.K., Andrés, J.A. (2013). Juxtaposition between host population structures: implications for disease transmission in sympatric cervid community. *Evolutionary Applications, 6,* 1001-1011.

Warren, D.L., Cardillo, M., Rosauer, D.F., Bolnick, D.I. (2014). Mistaking geography for biology: inferring processes from species distributions. *Trends in Ecology & Evolution, 29,* 572-580.

Weldenegodguad, M., Pokharel, K., Ming, Y., Honkatukia, M., Peippo, J., Reilas, T., Røed, K.H., Kantanen, J. (2020). Genome sequence and comparative analysis of reindeer (*Rangifer tarandus*) in northern Eurasia. *Scientific Reports, 10,* 8980.

Yannic, G., St-Laurent, M.-H., Ortego, J., Taillon, J., Beauchemin, A., Bernatchez, L., Dussault, C., Côté, S.D. (2016). Integrating ecological and genetic structure to define management units for caribou in Eastern Canada. *Conservation Genetics, 17,* 437-453.

Yannic, G., Ortego, J., Pellissier, L., Lecomte, N., Bernatchez, L., Côté, S.D. (2017). Linking genetic and ecological differentiation in an ungulate with a circumpolar distribution. *Ecography, 40,* 1-15.
